# Multiple interactions are involved in a highly specific association of the Mod(mdg4)-67.2 isoform with the Su(Hw) sites in *Drosophila*

**DOI:** 10.1098/rsob.170150

**Published:** 2017-10-11

**Authors:** Larisa Melnikova, Margarita Kostyuchenko, Varvara Molodina, Alexander Parshikov, Pavel Georgiev, Anton Golovnin

**Affiliations:** 1 Department of Drosophila Molecular Genetics, Institute of Gene Biology, Russian Academy of Sciences, 34/5 Vavilov St., 119334 Moscow, Russia; 2 Department of the Control of Genetic Processes, Institute of Gene Biology, Russian Academy of Sciences, 34/5 Vavilov St., 119334 Moscow, Russia

**Keywords:** transcription factors, chromatin insulators, BTB domain, CP190, FLYWCH domain, Mod(mdg4)

## Abstract

The best-studied *Drosophila* insulator complex consists of two BTB-containing proteins, the Mod(mdg4)-67.2 isoform and CP190, which are recruited to the chromatin through interactions with the DNA-binding Su(Hw) protein. It was shown previously that Mod(mdg4)-67.2 is critical for the enhancer-blocking activity of the Su(Hw) insulators and it differs from more than 30 other Mod(mdg4) isoforms by the C-terminal domain required for a specific interaction with Su(Hw) only. The mechanism of the highly specific association between Mod(mdg4)-67.2 and Su(Hw) is not well understood. Therefore, we have performed a detailed analysis of domains involved in the interaction of Mod(mdg4)-67.2 with Su(Hw) and CP190. We found that the N-terminal region of Su(Hw) interacts with the glutamine-rich domain common to all the Mod(mdg4) isoforms. The unique C-terminal part of Mod(mdg4)-67.2 contains the Su(Hw)-interacting domain and the FLYWCH domain that facilitates a specific association between Mod(mdg4)-67.2 and the CP190/Su(Hw) complex. Finally, interaction between the BTB domain of Mod(mdg4)-67.2 and the M domain of CP190 has been demonstrated. By using transgenic lines expressing different protein variants, we have shown that all the newly identified interactions are to a greater or lesser extent redundant, which increases the reliability in the formation of the protein complexes.

## 1. Introduction

The *mod*(*mdg4*) gene, also known as *E(var)3*-*93D*, encodes a large set of protein isoforms with specific functions in the regulation of the chromatin structure of different genes [1–6]. Protein isoforms produced by *mod(mdg4)* contain a common 402 aa N-terminal region encoded by the four 5′-exons, but differ in their C-terminal region encoded by alternative 3′-exons. Interestingly, all mRNAs for the alternative Mod(mdg4) isoforms are mainly produced by trans-splicing [7–10]. The Mod(mdg4) isoforms contain a BTB/POZ domain, an additional dimerization domain and a glutamine-rich (Q) region in the N terminus [1,11].

The BTB (bric-a-brac, tramtrack and broad complex)/POZ (poxvirus and zinc finger) domain is a conserved protein–protein interaction motif contained in a variety of transcription factors involved in development, chromatin remodelling, insulator activity and carcinogenesis [12,13]. All the well-studied mammalian BTB domains form obligate homodimers and, rarely, tetramers [13]. The BTB domain of Mod(mdg4) belongs to the ‘ttk group’ that contains several highly conserved sequences not found in other BTB domains [14,15]. The BTB domains of the ttk group can multimerize [14], which was suggested to be essential for the ability of the Mod(mdg4) isoforms to support pairing between the distantly located sites in the chromosomes [16].

Mutational dissection and differential binding of the Mod(mdg4) isoforms on polytene chromosomes suggest that the variable C-terminal regions encoded by the alternative 3′ exons determine their functional specificity [1,6,17]. The variable C-terminal regions interact specifically with different proteins [3,18,19]. So far, the functional roles of only two Mod(mdg4) isoforms have been studied in detail. The Mod(mdg4)-56.3/MNM (Modifier of Mdg4 in Meiosis) isoform is required for the homologue conjunction during meiosis [6,20], while the best-studied Mod(mdg4)-67.2 isoform is important for the enhancer-blocking activity of the Su(Hw) insulators [3,21,22]. Twelve repeated binding sites for the Su(Hw) constitute the best-studied *Drosophila* insulator, which was found at the 5′ regulatory region of the *gypsy* retrotransposon [23–25]. Insulators in the *Drosophila* and vertebrate genomes have been identified based on their ability to disrupt the communication between an enhancer and a promoter when inserted between them [26–34]. The *Drosophila* Suppressor of Hairy-wing (Su(Hw)) protein is a classical insulator protein that contains an array of 12 zinc fingers of the C2H2 and C3H types [23,35]. The C2H2 domains, from 5 to 10, specifically recognize an approximately 18 bp site [36]. Later, several other insulator proteins (dCTCF, Zw5, ZIPIC and Pita) with clusters of zinc finger domains have been identified [37–43].

The best described insulator found at the 5′ regulatory region of the *gypsy* retrotransposon has a unique structure because it consists of twelve repeating binding sites for Su(Hw) [23–25]. All other genomic regions contain only one or rarely two or three bindings sites for Su(Hw) [36,44]. At the same time, in the transgenic lines only four synthetic Su(Hw)-binding sites can function as an effective insulator [45], but the genomic regulatory elements containing one or two Su(Hw) sites also display strong enhancer-blocking activity [44,46–48]. This discrepancy might be explained by the existence of additional unknown insulator proteins that function in a cooperation with the Su(Hw). Thus, the gypsy insulator is an exceptional example of insulators consisting of the reiterated binding sites for only one protein.

In addition to Mod(mdg4)-67.2, the CP190 protein interacts directly with Su(Hw) and both are required for the activity of the Su(Hw)-dependent insulators [49,50]. In the genome-wide studies [44,51,52], three classes of the Su(Hw)-binding regions have been identified, which are characterized by the binding of the Su(Hw) alone (SBS-O), of both Su(Hw) and CP190 (SBS-C), and all the three proteins (SBS-CM) [44,53–55]. The stand-alone Su(Hw) sites (SBS-O) usually repress transcription [44], while SBS-CM sites display enhancer-blocking activity. In contrast to Mod(mdg4)-67.2, CP190 interacts beside the Su(Hw) with many other known insulator ZF proteins including Pita, dCTCF and ZIPIC [38,40,41,56]. The CP190 protein contains an N-terminal classical BTB/POZ domain that forms a homodimer that is involved in the interaction with dCTCF and Pita proteins [38,40]. CP190 preferentially binds near the transcription start sites of genes, suggesting a role of this protein in the organization of promoter architecture [44,57,58]. It was shown that CP190 participates in recruiting of the NURF, dREAM and SAGA complexes to chromatin [59–62], which are critical for the activity of promoters. Transcriptional complexes recruited to chromatin by the Mod(mdg4) isoforms have not been identified yet, but Mod(mdg4)-67.2 is essential for the enhancer-blocking activity of Su(Hw) [11,21,63]. For example, Mod(mdg4)-67.2 blocks the eye-specific enhancer by a direct interaction with Zeste that supports the enhancer-promoter communication of the *white* gene [22,64].

Here, we have studied how Mod(mdg4)-67.2 is specifically targeted to the Su(Hw)/CP190 complex. While CP190 also interacts with many other DNA-binding proteins, Mod(mdg)-67.2 interacts only with the Su(Hw). Previously, it was suggested that such specificity is dictated by an interaction between the unique part of the Mod(mdg4)-67.2 isoform and the C-terminal region of Su(Hw), between aa 716 and 892, named the Mod(mdg4)-67.2-interacting domain, MID [63,65]. Unexpectedly, we found that the *Su(Hw)*^e7^ mutant lacking the MID was still able to recruit Mod(mdg4)-67.2 to the Su(Hw) sites. For this reason, we re-examined the interactions between the insulator proteins and found new domains in these proteins that are essential for the specific recruiting of the Mod(mdg4)-67.2 to the Su(Hw) sites.

## 2. Material and methods

The constructs for the yeast two-hybrid assay, GST pull-down assay and transgenic constructs, and details of experimental and analytical procedures, are described in the electronic supplementary material.

### 2.1. *Drosophila* strains, germ line transformation and genetic crosses

The construct together with P25.7wc, a *P* element with defective inverted repeats used as a transposase source, was injected into *y ac w^1118^* preblastoderm embryos as described [66]. All flies were maintained at 25°C on the standard yeast medium. The resulting flies were crossed with *y ac w^1118^* flies, and the transgenic progeny were identified by their eye colour. Chromosome localization of various transgene insertions was determined by crossing the transformants with the *y ac w^1118^* balancer stock carrying dominant markers, *In(2RL),CyO* for chromosome 2 and *In(3LR)TM3,Sb* for chromosome 3. The generation of transgenic lines and construct introduction into the *mod(mdg4)^u1^* or *Su(Hw)^v^/Su(Hw)^e04061^* background were performed as described [21]. To express transgenes regulated by the UAS promoter, flies homozygous for the construct were crossed with the y1 w*; P{Act5C-GAL4}25FO1/CyO, y+ driver strain (Bloomington Center #4414).

The effects of Mod(mdg4) variants produced from homozygous expression vectors and various mutation combinations were scored by two researchers independently. The level of expression of *yellow* and *cut* phenotypes was evaluated in 3- to 5-day-old males developing at 25°C. For *yellow* phenotypes, wild-type expression in the abdominal cuticle, wings and bristles was assigned an arbitrary score of 5, while the absence of *yellow* expression was scored 1, using as reference the flies in which the *y* allele was characterized previously. Representative wing forms shown in the figures were selected as ‘average’ from the series of wings arranged in order of increasing severity of their mutant phenotype. At least 50 flies from each *y* line were scored.

### 2.2. Two-hybrid and *in vitro* interactions

Two-hybrid assays were carried out with yeast strain pJ694A using plasmids and protocols from Clontech (Palo Alto, CA). For growth assays, plasmids were transformed into yeast pJ694A cells by the lithium acetate method, as described by the manufacturer, and plated on media without tryptophan and leucine. After 3 days of growth at 30°C, the cells were plated on selective media without tryptophan, leucine, histidine and adenine, and their growth was compared after 2–3 days.

For GST pull-down experiments, GST-Mod(mdg4)-67.2, GST-CP190, GST-Su(Hw) or GST alone was incubated with Glutathione Sepharose 4B beads in binding buffer (20 mM Hepes-KOH (pH 7.6), 200 mM KCl, 2.5 mM MgCl_2_, 10% glycerol, 0.05% NP40) for 2 h. The beads were then blocked in 5% BSA for 1 h and incubated with 6His-tagged proteins for 3 h. After incubation, the beads were washed three times in wash buffer (10 mM Tris-HCl (pH 7.5), 1 mM EDTA, 0.2% NP40, 400 mM NaCl), boiled in Laemmli buffer and resolved in 8% SDS PAAG. The proteins were blotted onto a PVDF membrane, which was then incubated with antibodies to GST (Amersham) or His (Amersham).

### 2.3. RNA interference (RNAi) treatment and analysis of S2 cells in culture

CP190 cDNA templates were amplified by PCR using the primer pairs 5′-ATGGGTGAAGTCAAGTCCGTGAAAG-3′ and 5′-GAATTCCTTAACCTCTTCCAAAC-3′, with the 5′ end of each primer containing the T7 RNA polymerase promoter site. PCR products were purified using the Gel Extraction Kit (Zymo Research) as recommended by the manufacturer. Purified PCR products were used to produce double-stranded RNA (dsRNA) using a Megascript T7 transcription kit (Ambion). The RNA was purified according to the manufacturer's protocol, heated at 65°C for 30 min and left to cool at room temperature. Its samples were then resolved in agarose gel to test for the quality of dsRNA. *Drosophila* embryonic S2 cells were grown in Schneider's insect medium (Sigma) supplemented with 10% fetal calf serum (FCS, HyClone) at 27°C. The RNAi treatment and subsequent viable cell count analysis of S2 culture were basically performed as described [67]. To express the pAc5.1Su(Hw)^1-238^-FLAG construct, the S2 cells were transformed using the Effectene Transfection Reagent (Qiagen) as recommended by the manufacturer. Nuclear extracts were prepared and immunoprecipitation experiments were performed as described previously [68].

### 2.4. Protein extract preparation from males and co-IP analysis

The material (about 150–200 mg of adult males, sufficient for four or five independent immunoprecipitations) was homogenized in 5 ml of buffer IP-S+ (10 mM Tris-HCl (pH 7.5), 10 mM NaCl; 10 mM MgCl_2_; 1 mM EDTA; 1 mM EGTA; 1 mM DTT; 250 mM sucrose and PMSF, leupeten, pepstatinA) at +4C using a Douncer with a type A pestle. Then the homogenate was transferred through the BD Falcon filter to a 50 ml tube and spun down on a centrifuge for 5 min, at 4000*g* at 4°C. The supernatant was discarded. The pellet was resuspended in 3 ml of buffer IP-S+ and then spun down in the same way. This washing step was repeated 3 times. To the pellet, 0, 5 ml IP-10 buffer+ (10 mM Tris-HCl (pH 7.5), 10 mM NaCl, 10 mM MgCl_2_, 1 mM EDTA, 1 mM EGTA, 1 mM DTT, 0,1% NP-40, 10% glycerol and Roche Complete Protease Inhibitor Cocktail) was added and the pellet was homogenized at +4C using a Douncer with a type B pestle. Equal volume of IP-850 buffer (10 mM Tris-HCl (pH 7.5), 850 mM NaCl, 10 mM MgCl_2_, 1 mM EDTA, 1 mM EGTA, 1 mM DTT, 0.1% NP-40, 10% glycerol and Roche Complete Protease Inhibitor Cocktail) was added to the homogenate. It was mixed gently and left on ice from 30 min to 1 h. Then several lysate centrifugation steps were performed at maximum speed. Each time the lysate was transferred to a new tube without disturbing the pellet. Prior to immunoprecipitation, lysate was diluted three times in IP-0 buffer (10 mM Tris-HCl (pH 7.5), 10 mM MgCl_2_, 1 mM EDTA, 1 mM EGTA, 1 mM DTT, 0,1% NP-40, 10% glycerol and Roche Complete Protease Inhibitor Cocktail). After centrifugation at maximum speed, the supernatant was transferred to the new tube and immunoprecipitation experiments were performed as described previously [68].

### 2.5. Immunostaining

Squashed salivary gland specimens were prepared and stained with antibodies against Mod(mdg4)-67.2, FLAG, Su(Hw) and CP190 as described [69], and examined under a Leica TCS SP2 confocal microscope.

### 2.6. Chromatin preparation and ChIP analysis

Chromatin was prepared from the middle pupa stage as described previously [70]. The resulting chromatin preparation was used for ChIP experiments as described previously [51]. At least three independent biological replicates were made. Primer sequences used in PCR for ChIP analysis are shown in electronic supplementary material, table S1.

### 2.7. Antibodies

Specific antibodies and working dilutions were as follows: mouse anti-FLAG (1 : 300) from Sigma, and rat anti-CP190 (1 : 500), rabbit anti-Mod(mdg4)-67.2 (1 : 500), mouse anti-Mod-common (1 : 500) and rabbit anti N-terminal domain of Su(Hw) (1 : 200) raised in our laboratory and described previously [51,70]. Rabbit antibodies against the C-terminal domain of Su(Hw) (1 : 200) were kindly supplied by M. Erokhin. The secondary antibodies were Cy3-conjugated anti-rat (Jackson ImmunoResearch), FITC-conjugated anti-rabbit (Jackson ImmunoResearch) and Cy5-conjugated anti-mouse (Jackson ImmunoResearch) IgGs, all used at a 1 : 500 dilution.

## 3. Results

### 3.1. Role of the C-terminal domain in Su(Hw) interaction with the Mod(mdg4)-67.2 *in vivo*

The Su(Hw)^e7^ mutation was previously characterized and is generated by a C → T transition at base 3069 that leads to production of a truncated protein lacking the last 223 amino acids that contain the MID region required for the interaction with Mod(mdg4)-67.2 [63,65,71]. The level of Su(Hw)^e7^ expression is comparable with that of wild-type protein (electronic supplementary material, figure S1*a*).

**Figure 1.**
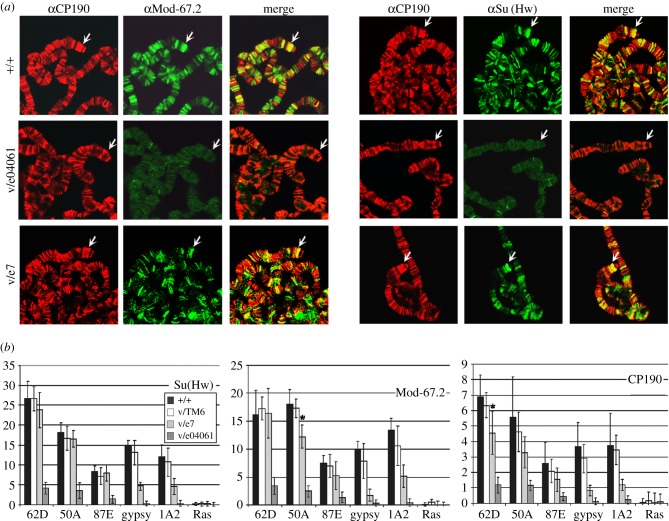
The role of the Su(Hw) C-terminal region in recruiting Mod(mdg4)-67.2 and CP190 to chromatin. (*a*) Polytene chromosomes from the salivary glands of third-instar *y^2^sc^D1^ct^6^* (+/+), *y^2^sc^D1^ct^6^*; *su(Hw)^v^/su(Hw)^e04061^* (v/e04061) and *y^2^sc^D1^ct^6^*; *su(Hw)^v^/su(Hw)^e7^* (v/e7) larvae co-stained with antibodies against the Mod(mdg4)-67.2 C-terminal region corresponding to the specific isoform (Mod-67.2, green) and CP190 (red) on the left or against Su(Hw) (green) and CP190 (red) on the right. Arrows indicate *gypsy* inserts at the tip of the X chromosome. (*b*) ChIP-qPCR analysis of Su(Hw), Mod(mdg4)-67.2 (Mod-67.2) and CP190 binding in middle pupae of the alleles +/+, v/e04061 and v/e7. Quantitative PCR (qPCR) was performed on the intergenic regions bound by Su(Hw). PCR products were amplified from two separate immunoprecipitates of three different chromatin preparations. The *ras64B* coding region (Ras) was used as a control devoid of Su(Hw)-binding sites. The per cent recovery of immunoprecipitated DNA (*Y* axis) was calculated relative to the amount of input DNA. Error bars indicate the standard deviation of three independent biological replicates. *p ≤ 0.05 (Student's *t*-test); in other cases, *p* ≤ 0.01.

As shown previously, Mod(mdg4)-67.2 protein completely co-localizes with Su(Hw) on polytene chromosomes [1,3]. The antibodies raised against the unique C-terminal domain of Mod(mdg4)-67.2 recognized about 200 sites on polytene chromosomes, in particular the sites corresponding to *gypsy* insertion in the *y^2^* mutation and the endogenous 1A2 insulator [3,46,48] at the tip of the X chromosome (figure 1*a*). In the *su(Hw)^–^* background (*su(Hw)^v^/su(Hw)^e04061^*), Su(Hw) proved to still strongly bind to several sites, which could be explained by a weak residual expression of the *su(Hw)^e04061^* allele generated by an insertion of the PiggyBac element near the start codon [72]. In *su(Hw)^–^* flies, almost no binding of Mod(mdg4)-67.2 to polytene chromosomes was observed, confirming the critical role of Su(Hw) in Mod(mdg4)-67.2 recruitment. Residual staining of Mod(mdg4)-67.2 at a few sites could be explained by a residual binding of the Su(Hw) to the same sites. Unfortunately, we were unable to directly test this point due to inability to independently examine the Su(Hw) and Mod(mdg4)-67.2 binding to the polytene chromosomes. The binding of CP190 was reduced only at a small number of sites, providing additional evidence that many different proteins recruit CP190 to the chromatin.

In *su(Hw)^v^/su(Hw)^e7^* larvae, the pattern of Su(Hw) binding to polytene chromosomes was the same as in wild-type larvae (figure 1*a*). Unexpectedly, we also found that a considerable number of Mod(mdg4)-67.2-positive sites coincided with sites for Su(Hw) and CP190. In particular, Mod(mdg4)-67.2 binds to the *y^2^* site at the tip of the X chromosome. Thus, the deletion of the Su(Hw) C-terminal domain only partially affects the recruitment of Mod(mdg4)-67.2 to the Su(Hw) sites.

To confirm the above results, we used ChIP to study the binding of insulator proteins with chromatin isolated from pupae expressing wild-type Su(Hw)+ (*su(Hw)^v^/TM6,Tb*), null for Su(Hw) (*su(Hw)^v^/su(Hw)^e04061^)* and Su(Hw)^e7^ (*su(Hw)^v^/su(Hw)^e7^*) (figure 1*b*; electronic supplementary material, figure S1*a*). To this end, we used the *gypsy* and four endogenous insulators that are bound by Su(Hw) in complex with C190 and Mod(mdg4)-67.2 (electronic supplementary material, figure S2) [47] and antibodies against the N-terminal domain of Su(Hw). In *su(Hw)^–^* pupae, we still observed residual Su(Hw) binding to strong insulator sites (50A, 62D), which was correlated with Mod(mdg4)-67.2 binding to the same sites. At the same time, the binding of Mod(mdg4)-67.2 and Su(Hw) to the *gypsy* and 1A2 insulators was almost completely absent. In *su(Hw)^v^/su(Hw)^e7^* pupae, ChIP analysis revealed almost normal Su(Hw) binding to 62D, 50A and 87E, while its binding to the *gypsy* and 1A2 insulators was reduced, suggesting that the C-terminal region contributes to association of Su(Hw) with chromatin. Similar results were obtained for Mod(mdg4)-67.2 and CP190. The direct correlation between the binding of Su(Hw), Mod(mdg4)-67.2 and CP190 suggests that the deletion of the C-terminal domain does not strongly affect the recruitment of Mod(mdg4)-67.2 and CP190 to the Su(Hw) sites. Thus, deletion of the MID only partially affects interaction of the truncated Su(Hw)e7 with Mod(mdg4)-67.2.

### 3.2. Identification domains responsible for the interaction of Mod(mdg4)-67.2 with Su(Hw) and CP190

**Figure 2.**
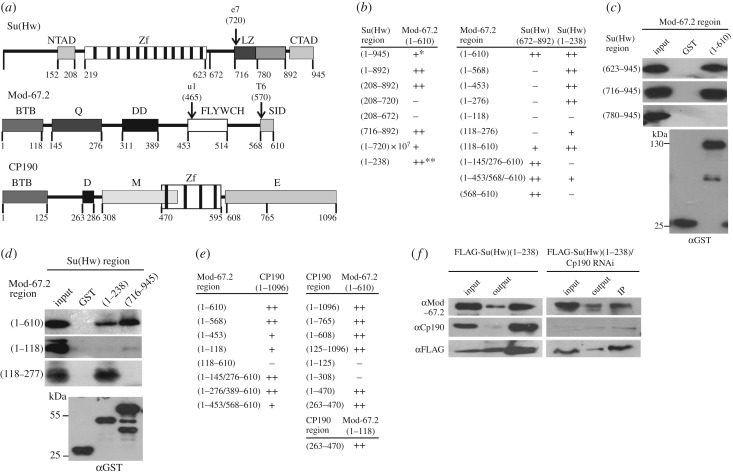
Summary of interactions between domains of Mod(mdg4)-67.2, Su(Hw) and CP190 proteins. (*a*) Structural scheme of Su(Hw), Mod(mdg4)-67.2 (Mod-67.2) and CP190 showing the domains of these proteins and the corresponding numbers of amino acid residues. Domain abbreviations: NTAD, N-terminal acidic domain; CTAD, C-terminal acidic domain; Zf, zinc-finger domains; LZ, leucine zipper motif; BTB, BTB/POZ domain; Q, glutamine-rich (Q-rich) region; DD, dimerization domain; FLYWCH, FLYWCH-type zinc finger domain; SID, Su(Hw) interaction domain; D, aspartic acid-rich (D-rich) domain; M, centrosomal targeting domain; E, C-terminal glutamic acid-rich domain. Arrows indicate the location of the *su(Hw)^e7^* (e7), *mod(mdg4)^u1^* (u1) and *mod(mdg4)^T6^* (T6) truncation alleles. (*b*) The results of testing Su(Hw) domains for the interaction with Mod-67.2 domains. All experiments were repeated in triplicate. Numbers in brackets are the numbers of amino acid residues. The plus signs indicate the relative strength of two-hybrid interaction (electronic supplementary material, figure S1*b*); the minus sign, the absence of the interaction; one asterisk, reduction in the interaction due to the repressive effect of the Su(Hw) C-terminal domain [73]; two asterisks, strong interaction observed only when DBD of GAL4 was fused to the C-terminal part of Su(Hw) derivatives. (*c*) Test for direct interaction between GST-fusion Mod(mdg4)-67.2 protein and His-fusion Su(Hw) domains in a GST pull-down experiment. (*d*) Test for direct interaction between GST-fusion Su(Hw) and His-fusion Mod(mdg4)-67.2 domains in a GST pull-down experiment. The interactions between the regions of insulator proteins were visualized by western blot analysis with anti-His tag monoclonal antibodies or with anti-GST antibodies used as loading control (at the bottom). The sample in the InPut lane contained 10% of His-fusion protein loaded onto Glutathione Sepharose together with GST-fusion insulator proteins. The sample in the GST column contained GST alone. Numbers in brackets are the numbers of amino acid residues. All results were reproduced in three independent experiments. (*e*) The results of testing Mod-67.2 domains for the interaction with CP190 domains. (*f*) Co-immunoprecipitation between the N-terminal domain of Su(Hw) fused to the FLAG epitope and insulator proteins under normal conditions and after CP190 RNAi treatment. The immunoprecipitated complexes were washed with 500 mM KCl-containing buffers before loading onto SDS-PAGE for western blot analysis with antibodies against the indicated proteins (CP190 or Mod-67.2) or the FLAG epitope. Input is the input fraction (10% of lysate used for immunoprecipitation); Output, the supernatant after immunoprecipitation; IP, the immunoprecipitate.

The unexpected recruitment of the Mod(mdg4)-67.2 to chromatin in the line expressing Su(Hw)^e7^ suggests an existence of unknown interactions between the proteins in the insulator complex. For this reason, we re-examined the domains of Su(Hw) involved in the interaction with Mod(mdg4)-67.2 using the yeast two-hybrid assay (figure 2*a*,*b*; electronic supplementary material, figure S3), the method based on fusion of the GAL4 activation and the DNA-binding domains to the N- or C-ends of the test protein, or its part.

The Su(Hw) protein (figure 2*a*) contains a cluster of 12 zinc-finger domains (between aa 219 and 623), the N-terminal acidic domain (between aa 152 and 208), the domain resembling leucine zipper (LZ, between aa 716 and 780) and the C-terminal acidic domain (between aa 892 and 945) [25,71]. Previously, we found that the C-terminal acidic domain of Su(Hw) partially represses transcription in yeast [11,73], complicating interpretation of the results obtained using this system. For this reason, in most of the experiments we used a truncated version of the Su(Hw) protein lacking the C-terminal domain from the 892 to 945 aa region. As shown by using yeast two-hybrid [65] and GST pull-down [63] assays, Mod(mdg4)-67.2 interacts with the C-terminal region of Su(Hw) between aa 716 and 892, including LZ. The Mod(mdg4)-67.2 protein interacts with Su(Hw) through the unique C-terminal domain (aa 453–610) that includes a FLYWCH-type zinc finger domain (between aa 453 and 514) (figure 2*a*).

The results confirmed previous data [63,65] that Mod(mdg4)-67.2 interacts with MID (the 716–892 region of Su(Hw), including LZ) (figure 2*b*). This interaction was also demonstrated in GST pull-down experiments (figure 2*c*). Unexpectedly, we observed that Mod(mdg4)-67.2 interacted with the N-terminal domain of Su(Hw) (aa 1–238) when the GAL4 domain was fused to the C-end of the latter protein. This interaction was confirmed in the GST pull-down assay with the bacterially expressed Su(Hw) N-terminal domain and the Mod(mdg4)-67.2 protein (figure 2*d*).

Next, we tested the domains of Mod(mdg4)-67.2 that are responsible for the interaction with Su(Hw) (figure 2*b*). In addition to the unique C-terminal region (aa 453–610), Mod(mdg4)-67.2 contains the BTB/POZ domain common to all its isoforms, a glutamine-rich (Q-rich) region and the dimerization domain (DD) [3,11] (figure 2*a*). Using a yeast two-hybrid assay, we tested Mod(mdg4)-67.2 with different deletions for the interaction with the N-terminal (aa 1–238) and C-terminal (aa 672–892) regions of the Su(Hw) protein. The 672–892 Su(Hw) fragment proved to interact with the Mod(mdg4)-67.2 derivative devoid of the FLYWCH domain (1–453/568–610) but not of the C-terminal region (1–568). Moreover, the 672–892 Su(Hw) fragment directly interacted with the Mod(mdg4)-67.2 C-terminal region (568–610) (figure 2*b*). Thus, the Su(Hw)-interacting domain of Mod(mdg4)-67.2 (SID) was narrowed down to the region between aa 568 and 610 (figure 2*a*,*b*).

The 1–238 region of Su(Hw) interacted with the C-terminal truncated variants of Mod(mdg4)-67.2, except for the variant that lacked the region of aa 145–277, including the Q-rich domain (figure 2*b*). The results of the GST pull-down assay confirmed the interaction between aa 1–238 of Su(Hw) and aa 118–277 of Mod(mdg4)-67.2 (figure 2*d*). Thus, a new interaction between the N-terminal domain of Su(Hw) and the Q-rich domain of Mod(mdg4)-67.2 was revealed.

As at most sites, CP190 and Mod(mdg4)-67.2 bind to Su(Hw) together [44], and CP190 also seems to contribute to specific recruiting of Mod(mdg4)-67.2. Indeed, the results of previous studies suggested that Mod(mdg4)-67.2 and CP190 interact with each other [11,50]. It was shown that the BTB domain of Mod(mdg4)-67.2 is required for interaction with CP190 [11]. Therefore, we tested different domains of these proteins in the yeast two-hybrid system in order to reveal and map the domains involved in their interaction (figure 2*e*). The CP190 protein contains several domains (figure 2*a*), including the BTB/POZ domain, aspartic acid-rich (D-rich) domain, four C2H2 zinc fingers and C-terminal glutamic acid-rich (E-rich) domain [57,74]. In addition to these motifs, CP190 also contains a centrosomal targeting domain (M) responsible for its localization to centrosomes during mitosis [75]. As a result, we found that the BTB domain of Mod(mdg4)-67.2 could interact with the M domain of CP190. However, deletion of the FLYWCH domain in Mod(mdg4)-67.2 somewhat reduced the strength of the signal, suggesting an auxiliary role for this domain in the CP190–Mod(mdg4)-67.2 interaction.

Finally, newly identified interaction between Mod(mdg4)-67.2 and the N-terminal domain of Su(Hw) was confirmed by *in vivo* testing for the interaction between the N-terminal region of Su(Hw) and Mod(mdg4)-67.2 in S2 cells (figure 2*f*). When the N-terminal region (aa 1–238) of Su(Hw) tagged with a triple FLAG epitope (FLAG-Su(Hw)1-238) was expressed in S2 cells, we observed co-immunoprecipitation between FLAG-Su(Hw)1-238 and endogenous Mod(mdg4)-67.2 (figure 2*f*). Taking into account that Mod(mdg4)-67.2 can directly interact with CP190, we examined the interaction of Mod(mdg4)-67.2 with the N-terminal region of Su(Hw) by co-immunoprecipitation with FLAG-Su(Hw)1-238 after RNAi-mediated knockdown of CP190 in S2 cells (figure 2*f*). Even in the absence of CP190 protein, Mod(mdg4)-67.2 was still precipitated together with FLAG-Su(Hw)1–238. Taken together, these results suggest that Mod(mdg4)-67.2 is able to interact with the N-terminal region of Su(Hw) *in vivo.*

### 3.3. Role of the N-terminal domain in Su(Hw) interaction with the Mod(mdg4)-67.2 and CP190 proteins *in vivo*

The obtained results suggest that the N-terminal domain of Su(Hw) can contribute to the recruitment of Mod(mdg4)-67.2 to the Su(Hw) sites. To test for the effect of the N-terminal deletion in Su(Hw), we produced transgenic lines expressing either the wild-type protein (Su(Hw)^+^) or its truncated variant (Su(Hw)ΔN 238-945) tagged with FLAG epitope under control of the *ubiquitin-63E* promoter and selected the lines in which the level of Su(Hw)ΔN or Su(Hw)+ expression in the *su(Hw)^−^* background (*su(Hw)^v^/su(Hw)^e04061^)* was comparable with that of the wild-type protein (electronic supplementary material, figure S1*b*).

**Figure 3.**
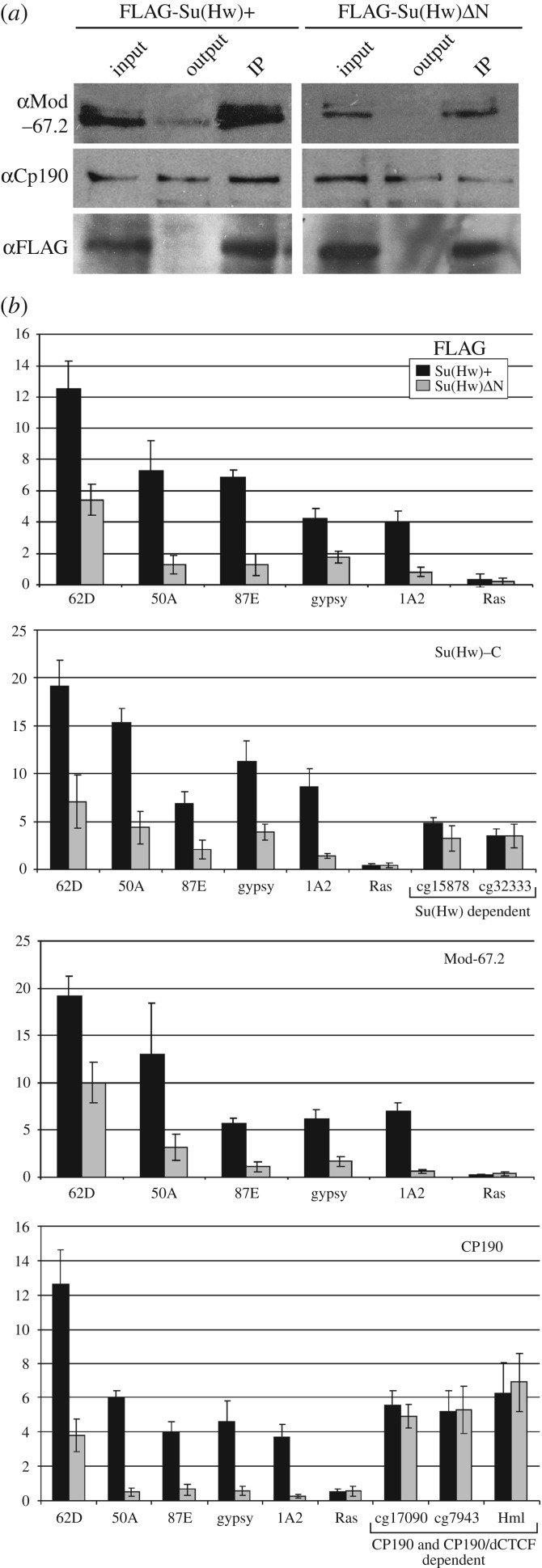
The role of the Su(Hw) N-terminal regions in recruiting Mod(mdg4)-67.2 and CP190 to chromatin. (*a*) Co-immunoprecipitation between the Su(Hw) variants and Mod(mdg4)-67.2 or CP190 proteins. All designations are as in figure 2*f*. (*b*) ChIP-qPCR analysis of Su(Hw), Mod-67.2 and CP190 binding in middle pupae of transgenic lines expressing Su(Hw)+ or Su(Hw)ΔN. PCR products were amplified from three separate immunoprecipitates of three different chromatin preparations. The *ras64B* coding region (Ras) was used as a control devoid of Su(Hw)-binding sites. Error bars indicate the standard deviation of three independent biological replicates. *p ≤ 0.01 (Student's *t*-test). Other designations are as in figure 1*b*. The experiments were performed in the *y^2^sc^D1^ct^6^*; *su(Hw)^v^/su(Hw)^e04061^* background. Transgene abbreviations: Su(Hw)+ – *P{w^+^;UbqW-Su(Hw)1-945-FLAG}/ P{w^+^;UbqW-Su(Hw)1-945-FLAG};* SuΔN – *P{w^+^;UbqW-Su(Hw)238-945-FLAG}/ P{w^+^;UbqW-Su(Hw)238-945-FLAG}*.

The interactions between the Su(Hw) variants and Mod(mdg4)-67.2 or CP190 were tested by co-IP in the extracts prepared from the 2-day-old males of the corresponding transgenic line (figure 3*a*). We observed a strong co-immunoprecipitation between the FLAG-Su(Hw)+ and Mod(mdg4)-67.2 or CP190. The Mod(mdg4)-67.2 was also precipitated by the Su(Hw) variant with deletions in the N-terminal domain.

The binding of the insulator proteins in pupae was analysed using ChIP analysis. In addition to the five Su(Hw)/Mod(mdg4)-67.2/CP190 sites, we tested two stand-alone Su(Hw) sites, two stand-alone CP190 sites and one site in which dCTCF is co-localized with CP190 (figure 3*b*; electronic supplementary material, figure S2). As it was impossible to detect Su(Hw)ΔN transgenes with the antibodies to the Su(Hw) N-terminal domain, we used antibodies raised against its C-terminal domains, along with the anti-FLAG antibodies. The binding of Su(Hw)ΔN to the Su(Hw)/Mod(mdg4)-67.2/CP190 sites was strongly reduced, comparatively to Su(Hw)+ (figure 3*b*). Interestingly, we did not observe such difference in the binding between the Su(Hw) variants in the case of the control stand-alone Su(Hw) sites (figure 3*b*). Thus, the N-terminal domain is essential for preferential recruitment of Su(Hw) only to the CP190/Mod(mdg4)-67.2 sites.

The Mod(mdg4)-67.2 and CP190 binding to the Su(Hw)/Mod(mdg4)-67.2/CP190 sites, but not to the control (stand-alone CP190 or dCTCF/CP190) sites in the Su(Hw)ΔN pupae, was strongly reduced, correlating with the low-level binding of Su(Hw). These results showed that the Mod(mdg4)-67.2 and CP190 proteins weakly, but still specifically, interact with Su(Hw) after deletion of the N-terminal Su(Hw) domain.

### 3.4. Experiments with a genetic model system confirm the role of multiple interactions between Mod(mdg4)-67.2 and Su(Hw) proteins

**Figure 4.**
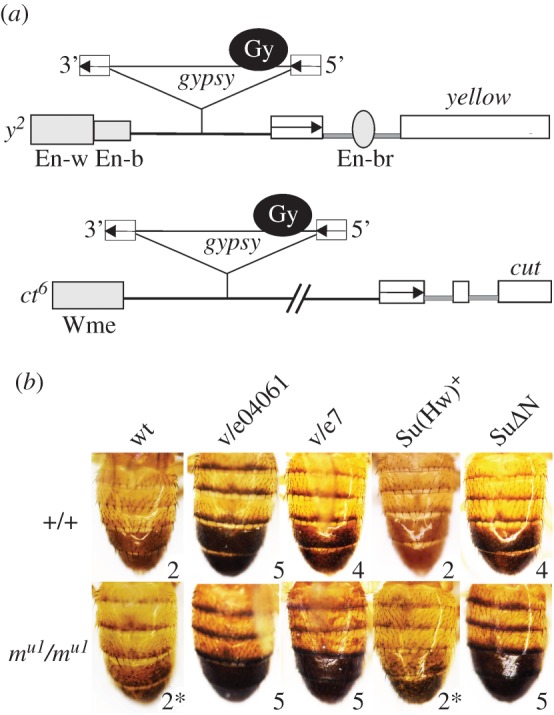
The functional role of multiple interactions between Mod(mdg4)-67.2 and Su(Hw) proteins. (*a*) Schemes (not to scale) of the *y^2^* and *ct^6^* alleles. Exons of the *yellow* and *cut* genes are shown as white rectangles; the *yellow* wing (En-w) and body (En-b) enhancers, as partially overlapping grey boxes; the bristle enhancer (En-br), as a grey oval in the *yellow* intron. The grey box (Wme) indicates the wing margin enhancer controlling *cut* expression in the wings. The transcription start sites are indicated by arrowheads. The *gypsy* insertions are shown as triangles in which the black circle marked Gy is the *gypsy* insulator and white boxes are long terminal repeats, with arrows indicating their direction. (*b*) Effect of Su(Hw) and its derivatives on the activity of the *gypsy* insulator in the *y^2^* allele in the *mod(mdg4)^+^* (+/+) or *mod(mdg4)^u1^* (*m*^*u1*^/*m*^*u1*^) background. The names of alleles included in analysis are listed at the top: wild-type (wt) – *y^2^sc^D1^ct^6^*, other designations are as in figures 1*a* and 3. Photos represent the abdominal pigmentation in 3-day-old males. Numbers indicate the scores of *yellow* expression in the body cuticle and wing blades, which ranged from 2 (pigmentation as in the *y^2^* allele) to 5 (pigmentation as in wild-type flies). Asterisks indicate mosaic abdomen specific for the *y^2^* allele on the *mod(mdg4)^u1^* background.

To determine the outcomes of mutations, we used *gypsy*-induced alleles in the *yellow* and *cut* loci. In the *y^2^* mutation (figure 4*a*), *gypsy* is inserted between the enhancers controlling *yellow* expression in the wings and body cuticle and the *yellow* promoter [24]. As a result, the Su(Hw) insulator blocks the wing and body enhancers, but not the bristle enhancer that is located in the *yellow* intron [24,76]. We also used four transgenic lines carrying a *gypsy* insertion between the *yellow* enhancers and the promoter, all of which displayed a y^2^-like phenotype (electronic supplementary material, figure S4*a*).

Inactivation of the Su(Hw) protein in the *su(Hw)^–^* background (*su(Hw)^v^/su(Hw)^e04061^*) completely restored *yellow* expression in the *y^2^* allele and transgenic lines, showing that the Su(Hw) protein is critical for insulation (figure 4*b*; electronic supplementary material, figure S4*b*). The *mod(mgd4)^u1^* mutation did not significantly change wing and body pigmentation of the *y^2^* allele (figure 4*b*) and *yellow* expression in transgenic lines (electronic supplementary material, figure S4*b*). In the *su(Hw)^v^/su(Hw)^e7^* background, *yellow* expression was only partially restored in the *y^2^* allele and transgenic lines (figure 4*b*; electronic supplementary material, figure S4*b*). Thus, the Su(Hw)^e7^ protein still weakly blocked the *yellow* enhancers. However, the combination of *su(Hw)^e7^* with *mod(mdg4)^u1^* led to a complete loss of enhancer-blocking activity in *y^2^* mutants and all transgenic lines, as in the *su(Hw)^–^* background. These results confirm that Mod(mdg4)-67.2 binds to the Su(Hw)^e7^ protein devoid of the C-terminal-interacting domain.

Likewise, the enhancer-blocking activity of the Su(Hw)+ and Su(Hw)ΔN proteins in the wild-type or *mod(mdg4)^u1^* background was compared by constructing transgenic lines carrying different combinations of mutations and transgenes (figure 4*b*; electronic supplementary material, figure S4*b*). In the lines expressing Su(Hw)+ protein, the *gypsy* insulator completely blocked the *yellow* enhancers. In contrast, Su(Hw)ΔN had only a partial effect on the *yellow* enhancer activity. The combination of Su(Hw)ΔN with *mod(mdg4)^u1^* resulted in complete restoration of enhancer activity, providing evidence for an additive effect of Mod(mdg4)-67.2 and mutant Su(Hw) protein. Thus, Su(Hw) with the deleted N-terminal domain is still able to recruit Mod(mdg4)-67.2.

### 3.5. Role of the C-terminal domain of Mod(mdg4)-67.2 in recruiting to the Su(Hw) sites *in vivo*

After identifying the Mod(mdg4)-67.2 domains involved in the interaction with Su(Hw) and CP190, our purpose was to test the role of these domains in recruiting Mod(Mdg4)-67.2 to chromatin *in vivo*. The aforementioned *mod(mdg4)^u1^* allele generates a mutant protein that lacks 148 aa corresponding to the unique C-terminal sequences (SID and FLYWCH) of Mod(mdg4)-67.2. The *mod(mdg4)^T6^* mutation results in the expression of mutant protein that lacks 43 C-terminal residues corresponding to the SID domain alone [65].

**Figure 5.**
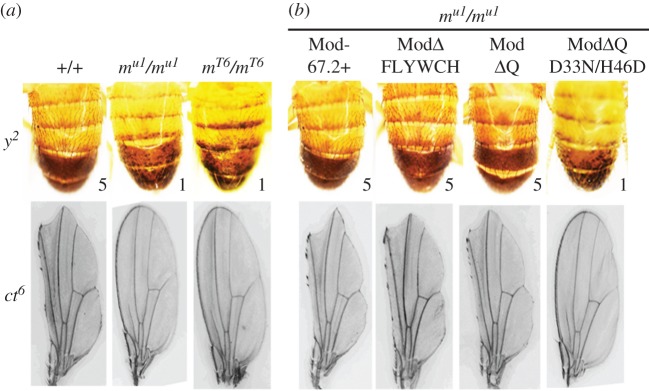
Effects of different Mod(mdg4) derivatives on the abdominal pigmentation in 3-day-old males in the *y^2^* allele and on the *cut* wing phenotype in the *ct^6^* allele. The genotypes of flies are indicated above the photos: (+/+)– *y^2^sc^D1^ct^6^*; (*m^u1^/ m^u1^*) – *y^2^sc^D1^ct^6^;mod(mdg4)^u1^/mod(mdg4)^u1^*; (*m^T6^/ m^T6^*) – *y^2^sc^D1^ct^6^;mod(mdg4)^T6^/mod(mdg4)^T6^*; (Mod-67.2+) – *P{w^+^;UAS-Mod-67.2}/P{Act5C-GAL4}25FO1*; (ModΔQ) – *P{w^+^;UAS-Mod*Δ*Q}/P{Act5C-GAL4}25FO1*; (ModΔFLYWCH) – *P{w^+^;UAS-Mod*Δ*FLYWCH}/P{Act5C-GAL4}25FO1*; and (ModΔQD33N/H46D) – double-mutant transgenic line ModΔQ with the most conserved aspartate (33) and histidine (46) in the Mod(mdg4)-67.2 BTB domain substituted by asparagine and acidic aspartate, respectively. Numbers show the scores of *yellow* expression in the bristles (1, loss of pigmentation; 5, wild-type pigmentation). Analysis of transgenic lines was performed in the *y^2^sc^D1^ct^6^*; *mod(mdg4)^u1^/mod(mdg4)^u1^* background.

In the *ct^6^* allele (figure 4*a*), *gypsy* is between the wing margin enhancer and the *cut* promoter, which are 85 kb apart [63]. The insulator in *ct^6^* completely blocks this enhancer, producing a cut wing phenotype. The *mod(mdg4)^u1^* and *mod(mdg4)^T6^* mutations affect the activity of the *gypsy* insulator inserted in the *y^2^* and *ct^6^* alleles (figure 5*a*). The *mod*(*mdg4*)*^u1^* and *mod*(*mdg4*)*^T6^* mutations almost completely suppress *ct^6^* phenotype, suggesting that Mod(mdg4)-67.2 is essential for the enhancer-blocking activity of the *gypsy* insulator in the case of the *ct^6^* allele. At the same time, the *mod*(*mdg4*) mutations enhance the mutant *y^2^* phenotype by repressing *yellow* expression in bristles and inducing a variegated pigmentation in the abdominal segments. Thus, binding of the Mod(mdg4)-67.2 protein prevents direct repression of the *yellow* promoter by the *gypsy* insulator in the *y^2^* allele.

**Figure 6.**
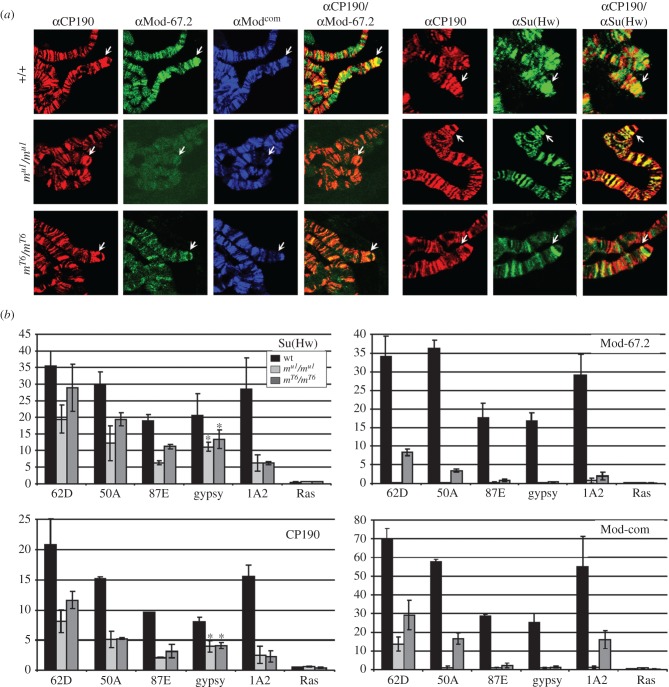
The role of the C-terminal sequences in recruiting Mod(mdg4)-67.2 to chromatin. (*a*) Polytene chromosomes of +/+, *m^u1^/ m^u1^*, and *m^T6^/ m^T6^* larvae. Polytene chromosomes stained with antibodies against CP190 (red), Mod-67.2 (green), Mod-com (common part of Mod(mdg4), blue) and N-terminal region of Su(Hw) (green). Analysis of transgenic lines was performed on the *y^2^sc^D1^ct^6^*; *mod(mdg4)^u1^/mod(mdg4)^u1^* background. Arrows indicate *gypsy* inserts at the X-chromosome tip. (*b*) ChIP-qPCR analysis of Su(Hw), Mod-67.2 and CP190 binding in middle pupae of *+/+*, *m^T6^/m^T6^* and *m^u1^/m^u1^* alleles. PCR products were amplified from two separate immunoprecipitates of three different chromatin preparations. The *ras64B* coding region (Ras) was used as a control devoid of Su(Hw)-binding sites. Error bars indicate the standard deviation of three independent biological replicates. *p ≤ 0.05 (Student's *t*-test); in other cases, *p* ≤ 0.01. Other designations are as in figure 1*b*.

We performed immunolocalization of these mutant proteins on polytene chromosomes (figure 6*a*) and analysed them by ChIP with chromatin from mutant pupae (figure 6*b*). The Mod(mdg4)^T6^ protein was detected with antibodies raised against the unique C-terminal domain (electronic supplementary material, figure S5), and the Mod(mdg4)^u1^ protein, with antibodies against the region common to all Mod(mdg4) isoforms.

ChIP analysis of mutant pupae showed that the Mod(mdg4)^u1^ protein did not bind to the selected Su(Hw) binding regions (figure 6*b*). In contrast to Mod(mdg4)^u1^, ChIP analysis showed that Mod(mdg4)^T6^ weakly binds to some Su(Hw) sites but not to the *gypsy* insulator in the *y^2^* allele. Faint bands of the Mod(mdg4)^T6^ protein were detected at relatively many sites on polytene chromosomes but not at the tip of the X chromosome corresponding to the *y^2^* allele (figure 6*a*). Thus, Mod(mdg4)^T6^ can weakly bind to the Su(Hw) sites. Both mutations, *mod(mdg4)^u1^* and *mod(mdg4)^T6^*, resulted in reduced binding of Su(Hw) and CP190, suggesting that Mod(mdg4)-67.2 facilitates the recruitment of Su(Hw) and CP190 to certain genomic regions.

These results showed that the SID domain is critical for effective recruitment of Mod(mdg4)-67.2 to the Su(Hw) sites.

### 3.6. Role of the Q-rich and BTB domains of Mod(mdg4)-67.2 in recruiting to the Su(Hw) sites *in vivo*

Our results and previous studies [63,65] demonstrated the crucial role of SID in Mod(mdg4)-67.2 recruitment to the Su(Hw) sites. However, the ability of Mod(mdg4)^T6^, in contrast to that of Mod(mdg4)^u1^, to weakly bind to some Su(Hw) sites suggests that the FLYWCH domain may also contribute to specific recruitment of Mod(mdg4)-67.2 to the Su(Hw) sites.

**Figure 7.**
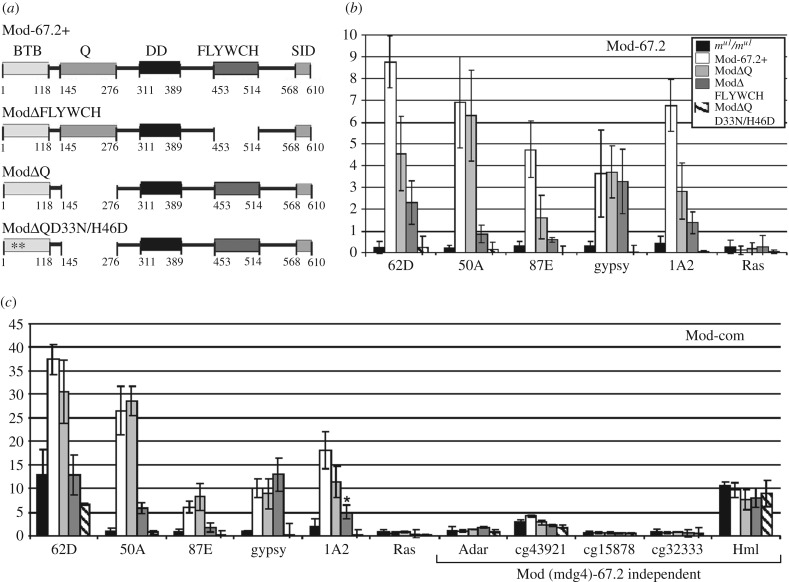
Role of FLYWCH and Q-rich domains in recruiting Mod(mdg4)-67.2 to chromatin *in vivo*. (*a*) Schemes of Mod(mdg4)-67.2 and its deletion derivatives: Mod-67.2+ is the wild-type protein, ModΔFLYWCH lacks the FLYWCH domain, ModΔQ lacks the Q-rich domain, and ModΔQD33N/H46D is a double mutant that lacks the Q-rich domain and has the most conserved aspartate (33) and histidine (46) in the BTB domain substituted by asparagine and acidic aspartate, respectively (indicated with asterisks). Other designations are as in figure 2*a*. (*b*,*c*) ChIP-qPCR analysis of Mod-67.2, and Mod-com binding in middle pupae of the above transgenic lines. PCR products were amplified from two separate immunoprecipitates of three different chromatin preparations. Error bars indicate the standard deviation of three independent biological replicates. *p ≤ 0.05 (Student's *t*-test), in other cases, *p* ≤ 0.01. The *ras64B* coding region (Ras) was used as a control devoid of Su(Hw)-binding sites. Other designations are as in figure 1*b*. Analysis of transgenic lines was performed in the *y^2^sc^D1^ct^6^*; *P{Act5C-GAL4}25FO1/+; mod(mdg4)^u1^/mod(mdg4)^u1^* background.

We assessed the role of FLYWCH and Q-rich domains in recruiting Mod(mdg4)-67.2 to chromatin. We used transgenic lines characterized by UAS-driven expression of Mod(mdg4)-67.2, Mod(mdg4)ΔQ and Mod(mdg4)ΔFLYWCH, in the *mod(mdg4)^u1^* background (figures 5*b* and 7*a–c*; electronic supplementary material, figure S5). To induce UAS expression, they were crossed with the transgenic *mod(mdg4)^u1^* line carrying the GAL4 gene under control of the *Act5C* promoter. Phenotypic analysis of the competence of mutant proteins in the insulator function was performed in male flies carrying *y^2^* and *ct^6^* mutations (figure 5*b*). The expression of Mod(mdg4)-67.2, Mod(mdg4)ΔQ and Mod(mdg4)ΔFLYWCH completely restored the mutant *mod(mdg4)^u1^* phenotype.

**Figure 8.**
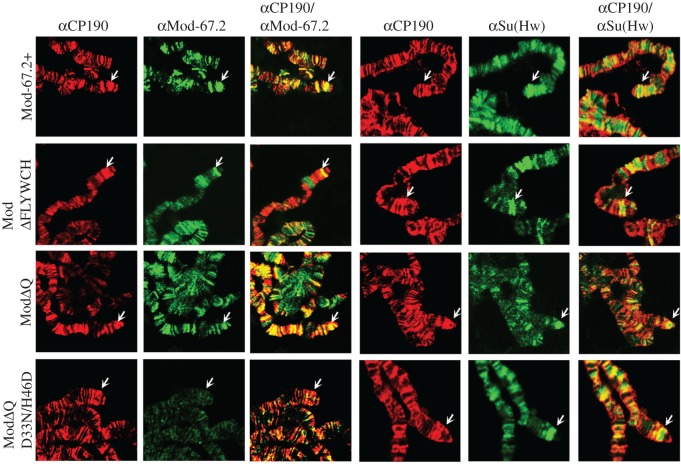
Immunostaining of polytene chromosomes from transgenic lines expressing Mod(mdg4)-67.2 derivatives. Polytene chromosomes of Mod-67.2+, ModΔFLYWCH, ModΔQ and ModΔQ D33N/H46D larvae stained with antibodies against CP190 (red), Mod-67.2 (green) and the N-terminal region of Su(Hw) (green). Analysis of transgenic lines was performed on the *y^2^sc^D1^ct^6^*; *mod(mdg4)^u1^/mod(mdg4)^u1^* background. Arrows indicate *gypsy* inserts at the tip of the X chromosome.

To test for the binding of Mod(mdg4)-67.2 variants to the Su(Hw) sites, we used ChIP at the pupa stage (figure 7*b*,*c*) and immunolocalization of proteins on polytene chromosomes (figure 8). To rule out non-specific effects of transgenic constructs, we tested the Su(Hw)/Mod(mdg4)-67.2/CP190 sites and Mod(mdg4)-independent sites (electronic supplementary material, figure S2). In ChIP with chromatin from pupae, Mod(mdg4)-67.2, Mod(mdg4)ΔQ and Mod(mdg4)ΔFLYWCH were found to bind to the test Su(Hw) sites (figure 7*b*,*c*). These proteins were also localized on the polytene chromosomes and the *y^2^* allele (figure 8). The Mod(mdg4) Δ FLYWCH was recruited to chromatin with lower efficiency than the Mod(mdg4)ΔQ or wild-type protein, suggesting a role for the FLYWCH domain in recruiting Mod(mdg4)-67.2 to the Su(Hw) sites. Deletion of the Q domain also slightly reduced the binding of the mutant Mod(mdg4)-67.2.

In our previous study [11], we made a double mutant Mod(mdg4)-67.2 protein, designated ModD33N/H46D, by substituting the most conserved aspartate (33) and histidine (46) in its BTB domain by asparagine and acidic aspartate, respectively. This mutant protein only weakly interacted with CP190 but still bound to the Su(Hw) sites and displayed normal functional activity. As the deletion of the Q domain only slightly affected the binding of the Mod(mdg4)ΔQ protein, we made a transgenic line expressing double mutant Mod(mdg4)ΔQ^D33N/H46D^ under control of the UAS promoter (figures 5*b* and 7*a–c*; electronic supplementary material, figure S5). The expression of ModΔQ^D33N/H46D^ did not complement the mutant *mod(mdg4)^u1^* phenotype (figure 5*b*). We also observed no binding of ModΔQ^D33N/H46D^ to the Su(Hw) sites in pupae analysed by ChIP (figure 7*b*,*c*) or to polytene chromosomes (figure 8). Thus, the combination of two mutations in ModΔQ^D33N/H46D^ resulted in the loss of the ability to bind to the Su(Hw) sites.

The binding of Su(Hw) and CP190 in ModΔQ^D33N/H46D^ pupae to the Su(Hw)/Mod(mdg4)-67.2/CP190 sites was reduced to the same extent as in the *mod(mdg4)^u1^* background (electronic supplementary material, figure S9). These results confirm that Mod(mdg4)-67.2 facilitates the recruitment of Su(Hw) and CP190 to chromatin.

## 4. Discussion

Our results suggest that multiple interactions are required for the formation of the Mod(mdg4)-67.2/CP190/Su(Hw) complex. It has been shown previously that the unique 567–610 region of the Mod(mdg4)-67.2 isoform interacts with the 693–880 region of Su(Hw), which is necessary for the enhancer-blocking activity [63,65]. However, deletion of the 224 C-terminal residues in Su(Hw)^e7^ only partially affects the Mod(mdg4)-67.2 recruitment, indicating that other domains may be involved in the interaction of these proteins. Interestingly, the Mod(mdg4)^T6^ protein lacking the 567–610 region required for interaction with Su(Hw) only weakly binds to the Su(Hw) sites. This suggests that the 567–610 region of Mod(mdg4)-67.2 may bind to an additional domain of Su(Hw). However, we failed to identify such a region in Su(Hw) or CP190. Alternatively, it is also possible that the 567–610 region of Mod(mdg4)-67.2 interacts with an unknown protein that also specifically associates with the Su(Hw). A further study is required to elucidate this question.

**Figure 9.**
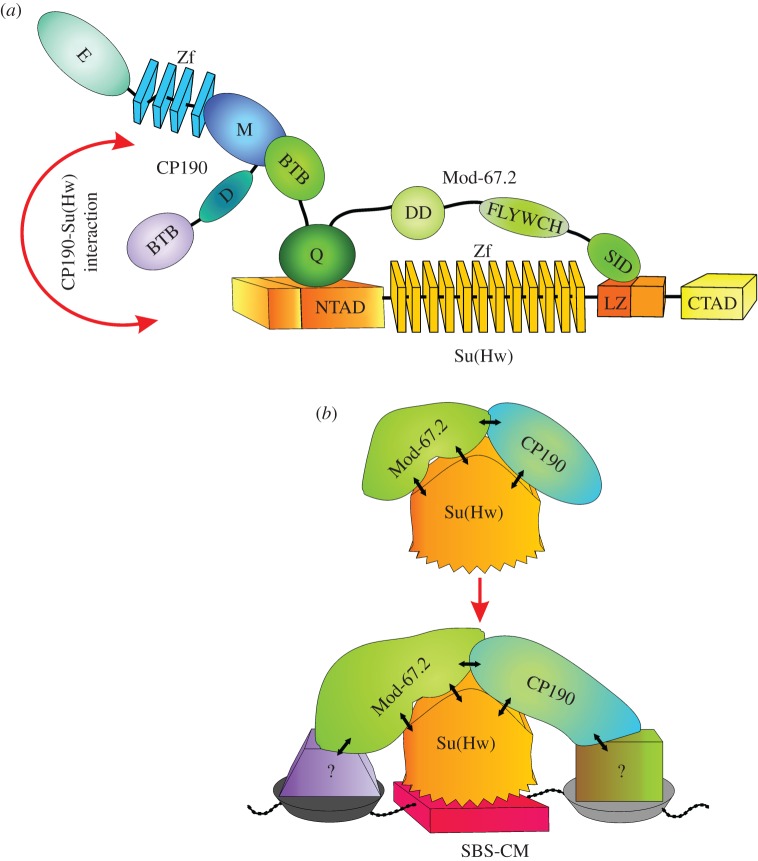
(*a*) A schematic of the protein–protein interactions involved in the formation of the Su(Hw)–CP190–Mod(mdg4)-67.2 complex. The arrow indicates a presumed direct interaction between Su(Hw) and CP190. Domain abbreviations are as in figure 2*a*. (*b*) A model proposing possible mechanisms of the Su(Hw) recruitment to the SBS-CM. The Su(Hw) complex is formed in the insulator bodies. The CP190 and Mod(mdg4)-67.2 interact with unknown DNA-binding proteins (?) in the SBS-CM that stabilize binding of the CP190-Mod(mdg4)-67.2-Su(Hw) complex to these genomic regions.

Here, we have found that the BTB and Q-rich domains of the Mod(mdg4)-67.2 (common to all its isoforms) interact with the M domain of CP190 and the N-terminal region of Su(Hw), respectively (figure 9*a*). As shown previously, the retention of the original Mod(mdg4) BTB domain in the Mod(mdg4)-67.2 isoform is critical for the specific recruitment of this protein to the Su(Hw)/CP190 sites [11]. For example, a chimeric Mod(mdg4)Gaf protein containing the GAF BTB domain can interact with Su(Hw) *in vitro* but completely loses its ability to associate with the Su(Hw)-binding regions [11]. Partially inactive BTB^D33N/H46D^ still shows a weak interaction with CP190, and ModD33N/H46D binds to the chromatin, similar to the wild-type protein [11]. However, here we have found that the double mutant carrying also the deletion of the Q domain fails to bind to the Su(Hw) sites. Thus, the Q-rich domain has a partially redundant role in recruiting Mod(mdg4)-67.2 to the chromatin. According to the genome-wide studies, all the Mod(mdg4)-67.2/Su(Hw) sites contain also the CP190 [44], suggesting that CP190 is important for the recruitment of Mod(mdg4)-67.2 to the Su(Hw) sites.

Our results also suggest a role for the FLYWCH domain in the specific Mod(mdg4)-67.2 recruitment to the Su(Hw)/CP190 sites. However, the mechanism of such an activity of the FLYWCH domain is still unknown. The results of the yeast two-hybrid assay show only that this domain improves the interaction between the BTB domain of Mod(mdg4)-67.2 and the M domain of CP190. Further analysis is required to resolve the mechanistic role of the FLYWCH domain in the functionality of Mod(mdg4)-67.2, taking into account that different variants of FLYWCH are present at the specific C-termini of the majority of the Mod(mdg4) isoforms [1].

Previously, we and others have shown that CP190, Mod(mdg4)-67.2 and Su(Hw) are co-localized in the nuclear speckles, named also the insulator bodies [51,70,77–80]. According to the current model [70,78], the insulator bodies help to form protein complexes that subsequently bind to the regulatory elements such as insulators and promoters. It could be possible that the Su(Hw)/CP190/Mod(mdg4)-67.2 complexes are performed in the insulator bodies, and after this are recruited to the chromatin. CP190 and Mod(mdg4)-67.2 might determine the recruitment of the insulator complexes to the specific sites, due to the assembly of the multiple protein–protein interactions. In accordance with this model, we found that the interaction of the Su(Hw) with CP190 and Mod(mdg4)-67.2 is essential for the recruitment of the insulator complex to SBS-CM (the Su(Hw)/CP190/Mod(mdg4)-67.2 sites) (figure 9*b*).

Taken together, it seems likely that the recruitment of the Mod(mdg4)-67.2, CP190 and Su(Hw) proteins to SBS-CM is mutually dependent. The specificity of the Mod(mgd)4-67.2 recruitment is achieved through complex interactions of the Mod(mdg4)-67.2 SID, FLYWCH and BTB domains with CP190 and Su(Hw). The existence of SBS-C, lacking Mod(mdg4)-67.2, might be explained by the masking of the CP190 M domain by proteins such as ZIPIC [40] at some genomic regions, which prevents the association of the CP190 M domain with the Mod(mdg4)BTB domain and a subsequent Mod(mdg4)-67.2 recruitment to the CP190/Su(Hw) sites.

The question remains unresolved as to why the other Mod(mdg4) isoforms do not bind to the Su(Hw) complex even though their common BTB and Q domains interact with the CP190 and Su(Hw) proteins, respectively. It seems likely that each Mod(mdg4) isoform specifically interacts with one or several DNA-binding transcription factors, as does Mod(mdg4)-67.2 with Su(Hw). If so, all the Mod(mdg4) isoforms prefer to interact with their specific protein complexes but not with the Su(Hw)–CP190 complex.

In summary, our results provide evidence for the high complexity of interactions between insulator proteins that are required to form the specific Su(Hw) insulator complex. Deletion of a single domain involved in the protein–protein interactions in either the Su(Hw) or the Mod(mdg4)-67.2 only partially disturbs its formation, indicating that the stability of the complex is ensured by the multiplicity/redundancy of such interactions.

## Supplementary Material

Supplementary materials: Figs S1 - S6, methods and supplementary table

## Data accessibility

All data supporting this article have been uploaded as the electronic supplementary material.
