# Supplementary material for: Multiple interactions are involved in a highly specific association of the Mod(mdg4)-67.2 isoform with the Su(Hw) sites in Drosophila
Source: Open Biol. 2017 Oct 11;7(10):170150. doi: 10.1098/rsob.170150 (PMC5666082; doi:10.1098/rsob.170150)
Supplement: Supplementary materials: Figs S1 - S6, methods and supplementary table [file rsob170150supp1.pdf]

Supplementary Figures

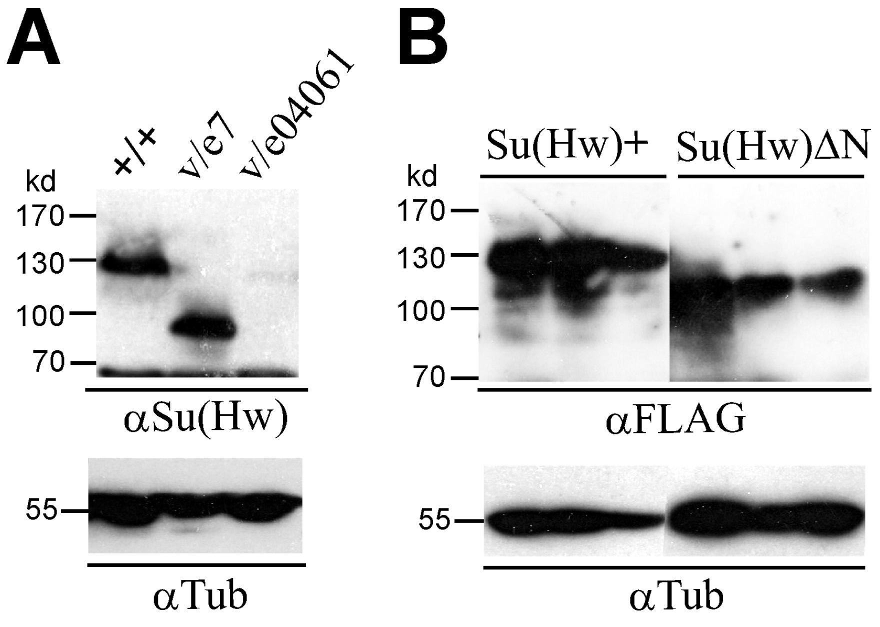

Figure S1. Western blot analysis of the expression of Su(Hw) protein and its derivatives *in vivo*. Su(Hw) isolated from adult flies as described (Gdula et al., 1997) was resolved by electrophoresis in 7.5% SDS-PAAG, electroblotted onto a PVDF membrane, and probed with antibodies against Su(Hw) or FLAG epitope (designated αSu(Hw) and αFLAG, respectively). Anti-tubulin staining (αTub) was used as loading control. The name of alleles included in analysis are indicated above the figure:

(A)[+/+] – *su(Hw)*$^{+}$*/su(Hw)*$^{+}$; [v/e7] – *su(Hw)*^{v}*/su(Hw)*^{e7}; [v/e04061] – *su(Hw)*^{v}*/su(Hw)*^{e04061};

(B) [Su(Hw)+] – *P{w*$^{+}$*;UbqW-Su(Hw)1-945-FLAG}/ P{w*$^{+}$*;UbqW-Su(Hw)1-945-FLAG};*

[Su(Hw)ΔN] – *P{w*$^{+}$*;UbqW-Su(Hw)238-945-FLAG}/ P{w*$^{+}$*;UbqW-Su(Hw)238-945-FLAG}*;

Expression analysis of transgenic lines was performed on the *y*^{2}*sc*^{D1}*ct*^{6}; *su(Hw)*^{v}*/ su(Hw)*^{e04061} background.

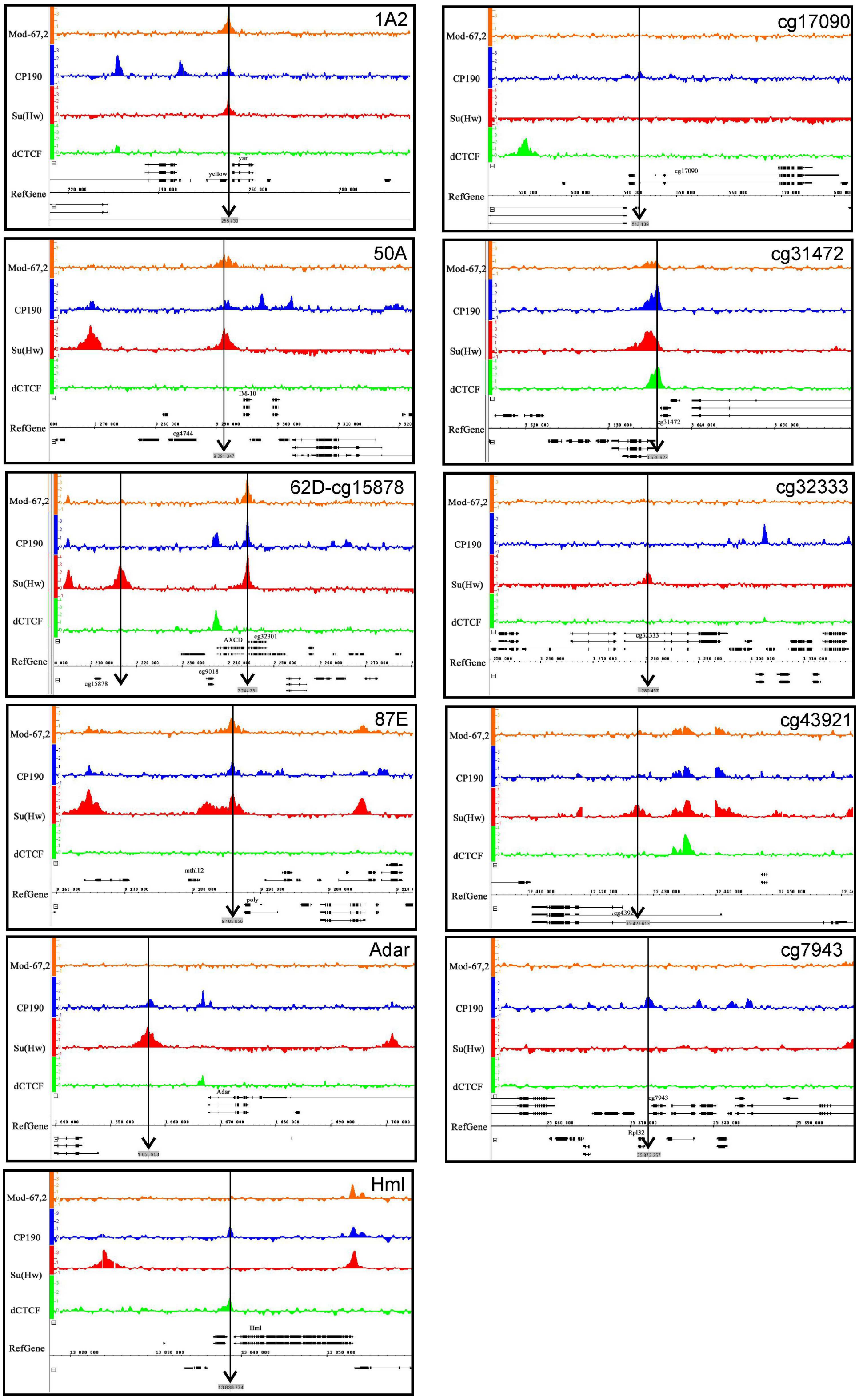

Figure S2. Genome browser view of insulator protein binding. The regions used in qPCR are indicated by vertical black arrows and numbered according to their chromosome position (FlyBase, 2006). Names of the identified genomic regions are given in the top right corner.

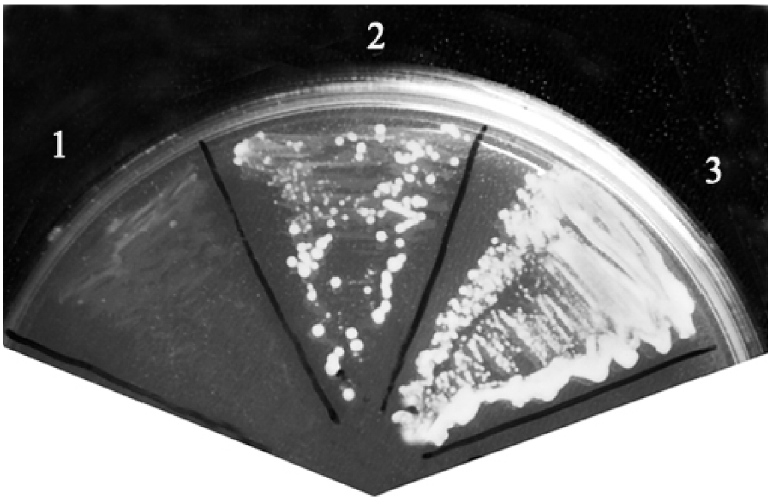

Figure S3. Test for two-hybrid interaction strength: (1) no interaction « - », (2) « + », (3) « ++ ».

A

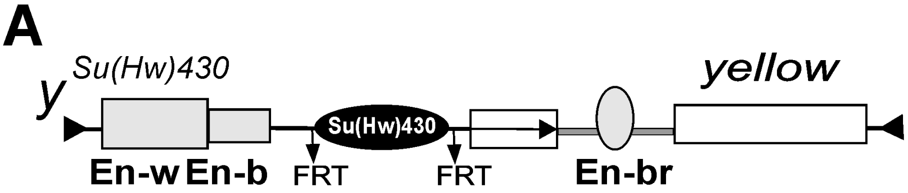

B

wing/body pigmentation

	5	4	3	2	N/T
+/+	-	-	-	4	4
m^{u1}/m^{u1}	-	-	-	4*	4/4
v/e7	-	4	-	-	4/4
v/e7;m^{u1}/m^{u1}	3	1	-	-	3/4
v/e04061	3	1	-	-	4/4
v/e04061;m^{u1}/m^{u1}	3	1	-	-	0/4
Su(Hw)+	-	-	-	4	4/4
Su(Hw)+;m^{u1}/m^{u1}	-	-	-	4*	4/4
Su(Hw)ΔN	-	2	2	-	4/4
Su(Hw)ΔN;m^{u1}/m^{u1}	3	1	-	-	4/4

Figure S4. The functional role of multiple interactions between Mod(mdg4)-67.2 and Su(Hw) proteins.

(A) Schemes (not to scale) of $Su(Hw)^{430}$ transgenic construct. Exons of the *yellow* gene are shown as white rectangles; the *yellow* wing (En-w) and body (En-b) enhancers, as partially overlapping gray boxes; the bristle enhancer (En-br), as a gray oval in the *yellow* intron. The *yellow* transcription start site is indicated by an arrowhead. Downward arrows indicate FRT target sites for Flp recombinase; the black oval between them (marked Su(Hw)430) is the insertion of the *gypsy* insulator in the construct.

(B) Effect of Su(Hw) and its derivatives on the activity of the *gypsy* insulator in different genomic positions in the wild-type or $mod(mdg4)^{u1}$ background. The names of alleles included in analysis are listed in the left column: [+/+] – $y^{2}sc^{D1}ct^{6}$; [m^{u1}/m^{u1}] –$mod(mdg4)^{u1}/mod(mdg4)^{u1}$; [v/e04061] – $su(Hw)^{v}/su(Hw)^{e04061}$; [v/e7] – $su(Hw)^{v}/su(Hw)^{e7}$; [v/e04061] – $su(Hw)^{v}/su(Hw)^{e04061}$; [Su(Hw)+] – *P{*$w^{+}$*;UbqW-Su(Hw)1-945-FLAG}/P{*w^{+}*;UbqW-Su(Hw)1-945-FLAG};* [Su(Hw)ΔN] –

P{w^+;UbqW-Su(Hw)238-945-FLAG}/P{w^+;UbqW-Su(Hw)238-945-FLAG}. Analysis of transgenic lines was performed on the $y^2sc^{D1}ct^6;su(Hw)^v/su(Hw)^{e04061}$ and $y^2sc^{D1}ct^6; su(Hw)^v mod(mdg4)^{u1}/ su(Hw)^{e04061} mod(mdg4)^{u1}$ backgrounds, respectively. N is the number of lines in which flies acquired a new *yellow* phenotype, compared to the control; T is the total number of lines examined. The scores of *yellow* expression in the body cuticle and wings blades ranged from 2 (pigmentation as in y^2 allele) to 5 (pigmentation as in wild-type flies). Asterisks indicate mosaic abdomen pigmentation specific for y^2 allele on the $mod(mdg4)^{u1}$ background. Arrows on the right of the column show the order in which introduction of new mutation combinations were made.

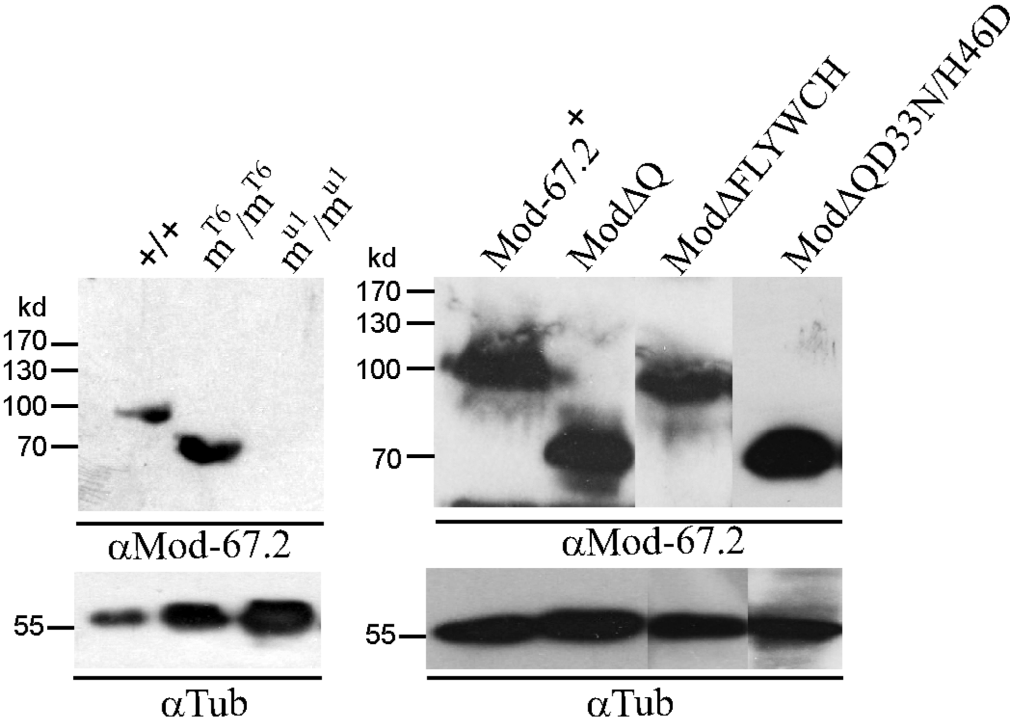

Figure S5. Western blot analysis of the expression of Mod(mdg4)-67.2 protein and its derivatives *in vivo*.

Mod(mdg4)-67.2 was isolated from adult flies as described (Gdula et al., 1997), resolved by electrophoresis in 7.5% SDS-PAGE, electroblotted onto a PVDF membrane, and probed with antibodies against the C-terminal region corresponding to the specific Mod(mdg4)-67.2 isoform (αMod-67.2) or FLAG epitope (αFLAG). Anti-tubulin staining (αTub) was used as loading control. The names of alleles included in analysis are indicated above the figure: [+/+] – $y^2sc^{D1}ct^6$; [m^{T6}/ m^{T6}] – $y^2sc^{D1}ct^6$; *mod(mdg4)*^{T6}/*mod(mdg4)*^{T6}; [m^{u1}/ m^{u1}] – $y^2sc^{D1}ct^6$; *mod(mdg4)*^{u1}/*mod(mdg4)*^{u1}; [Mod-67.2+] – *P{w*$^+$*;UAS-Mod-67.2}/P{Act5C-GAL4}25FO1*; [ModΔQ]– *P{w*$^+$*;UAS-ModΔQ}/P{Act5C-GAL4}25FO1*; [ModΔFLYWCH] – *P{w*$^+$*;UAS-ModΔFLYWCH}/P{Act5C-GAL4}25FO1*; and [ModΔQ D33N/H46D]– double-mutant transgenic line ModΔQ with the most conserved aspartate (33) and histidine (46) in the Mod(mdg4)-67.2 BTB domain substituted by asparagine and acidic aspartate, respectively. Expression analysis of transgenic lines was performed on the $y^2sc^{D1}ct^6$; *mod(mdg4)*^{u1}/*mod(mdg4)*^{u1} background.

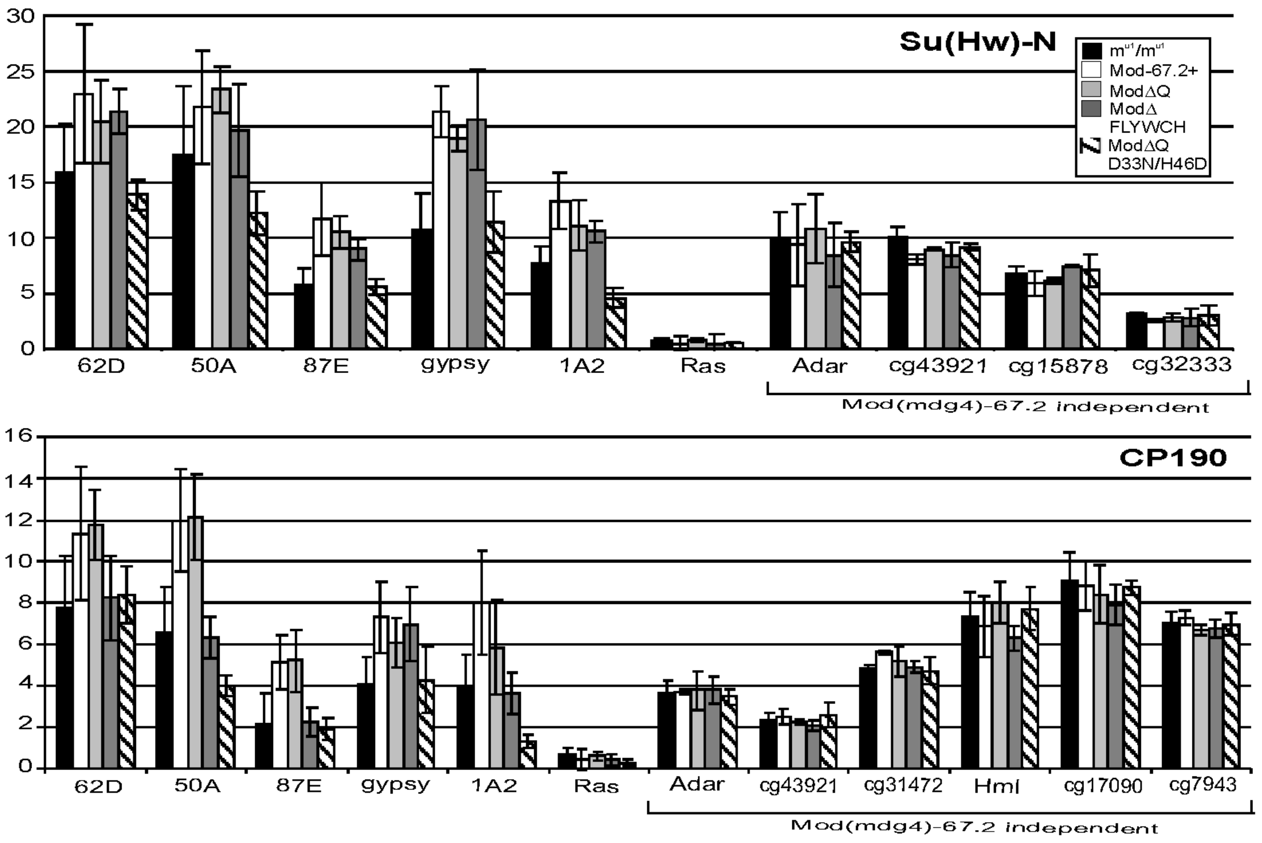

Figure S6. Role of FLYWCH and Q rich domains in recruiting Su(Hw) and CP190 to chromatin *in vivo*.

Analysis of transgenic lines was performed in the $y^{2}sc^{D1}ct^{6}$; *P{Act5C-GAL4}25FO1/+*; $mod(mdg4)^{u1}/mod(mdg4)^{u1}$ background. PCR products were amplified from two separate immunoprecipitates of three different chromatin preparations. ChIP was performed with antibodies against the Su(Hw) N-terminal region, and CP190. The *ras64B* coding region (Ras) was used as a control devoid of Su(Hw) binding sites. The percent recovery of immunoprecipitated DNA (Y axis) was calculated relative to the amount of input DNA. Error bars indicate standard deviation of three independent biological replicates.

Material and methods

Constructs for yeast two-hybrid assay

The coding region of Mod(mdg4)-67.2 from pGEX2T (kindly provided by D. Dorsett) was cloned in vectors pGBT9 or pGDA (plasmid that allows producing protein fusions with the activation domain of Gal4 on the C-terminal part (Golovnin et al. 2008) using EcoR1 and BamH1 sites (**pGBT9Mod-67.2 and pGDAMod-67.2**).

To prepare **Mod-67.2**$^{\mathbf{1\text{-}276}}$, PCR amplification was performed from pGBT9Mod-67.2 using primers 5-ataggatccttgcggcacaagttg-3' (containing BamHI site) and GAL DB. The PCR product was digested with EcoRI and BamHI and cloned in either pGBT9 or pGDA vector. The same strategy was used to generate **Mod-67.2**$^{\mathbf{1\text{-}568}}$ (5-ataggatccaatctcaaactcctcg-3' containing BamHI site), **Mod-67.2**$^{\mathbf{1\text{-}453}}$ (5-ataggatccggcgtacaccgcaggct-3' containing BamHI site) and **Mod-67.2**$^{\mathbf{1\text{-}118}}$ (5-ataggatccatccgttagccccttgat-3' containing BamHI site).

To prepare **Mod-67.2**$^{\mathbf{118\text{-}610}}$, PCR amplification was performed from pGBTMod-67.2 using 5-agaattcaatgtgcaaatcaaggggctaa-3' primer containing EcoRI site and plasmid reverse primers. The PCR product was digested with EcoRI and BamHI enzymes and cloned in either pGBT or pGDA vector.

To generate deletion of Q-rich domain (**Mod-67.2**$^{\mathbf{1\text{-}145/276\text{-}610}}$), pGBTMod-67.2 or pGDA Mod-67.2 was digested with BlpI, and the resulting plasmid was self-ligated. Deletion of DD domain (**Mod-67.2**$^{\mathbf{1\text{-}276/389\text{-}610}}$) was done by PCR amplification (primers 5'-ataaggcctgggcaattccatggggag-3' and 5'-ataaggcctgtcgacaccagcggg-3') followed by StuI digestion and self-ligation of the resulting plasmid.

To generate deletion of FLYWCH domain (**Mod-67.2**$^{\mathbf{1\text{-}453/568\text{-}610}}$), pGBTMod-67.2 or pGDA Mod-67.2 was digested with Eco72I and BstEII, and the resulting plasmid was self-ligated.

Mod-67.2$^{\mathbf{568\text{-}610}}$ plasmid was generated by PCR amplification of the corresponding fragment using 5'-gaattcatgctcgaggaagccgacgac-3' with inserted EcoRI site and ATG codon and a reverse plasmid primer.

To obtain **Mod-67.2**$^{\mathbf{118\text{-}276}}$ plasmids, PCR fragment from Mod-67.2 cDNA with attached EcoRI on 5' end (5' – GAATTCAAGATGCTAACGGATAAC - 3') and BamHI on 3' end (5' – GGATCCCTCGGCATCGCCGG - 3') was digested with these ensymez and cloned either in pGBT or in pGDA vectors digested with EcoRI and BamHI.

To generate **pGBT9Su(Hw)** plasmid, Su(Hw) cDNA was PCR-amplified from pGEM3ZfSu(Hw) with primers 5'-aatgagtgcctccaaggagggc-3' (upstream) and 5'-

ccgtcgactcaagctttctcttgttc-3' (downstream, containing SalI site). The PCR product was digested with SalI and cloned in the pGBT9 vector cleaved with SmaI and SalI.

To generate **pGDASu(Hw)** plasmid, cDNA of Su(Hw) was PCR-amplified from pGEM3ZfSu(Hw) with primers 5'-aatgagtgcctccaaggagggc-3' (upstream) and 5'-aaccatggaagctttctcttgttc-3' (downstream, containing NcoI site). This PCR product was digested with NcoI and cloned in pGDA cleaved with HpaI and NcoI.

To generate **pGBT9Su(Hw)ΔC** plasmid, Su(Hw) lacking the C-end (1–892) was PCR-amplified as described above, but the next downstream primer was 5'-tttgtcgacttcgcctgtgac-3'(with SalI site). The PCR product was digested with SalI and cloned in pGBT9 cleaved with SmaI and SalI.

To generate **pGDASu(Hw)ΔC** plasmid, we used the same strategy, but the next downstream primer was 5'-tttccatggttcgcctgtgac-3'(with NcoI site).

To generate **pGBT9Su(Hw)**$^{208\text{-}892}$, we PCR-amplified the corresponding fragment with 5'-aatgaactctagccagacaaagatc-3' and plasmid reverse primers. The PCR product was digested with SalI and cloned in pGBT9 cleaved with SmaI and SalI.

pGBT9 Su(Hw)$^{208\text{-}720}$ plasmid was generated by subcloning the EcoRI–EcoRI fragment from the previous plasmid in pGBT9 cleaved with EcoRI.

pGBT9 Su(Hw)$^{1\text{-}720}$ was generated by cloning the EcoRI–EcoRI fragment from pGBT9Su(Hw) in pGBT9 cleaved with EcoRI.

pGBT9Su(Hw)$^{208\text{-}672}$ was generated by PCR-amplification of the corresponding fragment with 5'-acgtcgactacaacctcctccgattcg-3' (containing SalI site) and GAL DB primers from pGBTSu(Hw)$^{208\text{-}720}$ plasmid. The PCR product was digested by EcoRI and SalI and cloned in pGBT9 cleaved with the same enzymes.

To obtain **Su(Hw)**$^{716\text{-}892}$ plasmids, PCR fragment from Su(Hw) cDNA with attached EcoRI on 5’ end (5’ – GAATTCCTCCTCCTTCTTCGCCT - 3’) and SalI on 3’ end (5’ – GTCGACTTCGCCTGTGAC - 3’) was digested with these ensymez and cloned either in pGBT or in pGDA vectors digested with EcoRI and SalI.

pGBT9Su(Hw)$^{672\text{-}892}$ was generated by PCR-amplification of the corresponding fragment from pGBT9Su(Hw)ΔC using 5'-caaagaaggacatcgaatcggag-3' and reverse plasmid primers. The PCR product was digested with SalI and cloned in pGBT9 cleaved with SmaI and SalI.

To generate **pGBT9Su(Hw)**$^{1\text{-}238}$, the EcoRI–XbaI (Klenow-filled) fragment from pGBT9Su(Hw) was cloned in pGBT9 cleaved with EcoRI–BamHI (filled in with Klenow fragment).

To generate **pGDASu(Hw)$^{1-238}$** or **pATGBSu(Hw)$^{1-238}$** (plasmid that allows producing protein fusions with the DNA binding domain of Gal4 on the C-terminal part (Mazur et al., 2005)), the EcoRI–BamHI fragment from **pGBT9Su(Hw)$^{1-238}$** was cloned in pGDA or pATGB cleaved with the same enzymes.

pGBT9Su(Hw)$^{208-945}$ was generated by PCR amplification of the corresponding fragment by 5'-aatgaactctagccagacaaagatc-3' and reverse plasmid primers. The PCR product was digested with SalI and cloned in vector pGBT9 digested with SmaI and SalI.

To generate **CP190$^{1-1096}$** plasmids, PCR fragment from CP190 cDNA with attached EcoRI on 5’ end (5’ –GAATTCATGGGTGAAGTC- 3’) and BglII on 3’ end (5’ –AGATCTTAGTAAATATAGCTCCTC- 3’) was digested with these enzymes and 741bp EcoRI-EcoRI fragment was cloned in either pGBT or pGDA vectors digested with EcoRI. Orientation of insertion was verified by RCR. PCR fragment with full-length CP190 cDNA was once again digested by NruI and BglII. Then resulted fragment 2800bp was ligated in the obtained from the first step plasmids which were digested by NruI and BamHI. Resulted plasmid contain full-length CP190 cDNA in frame with either GAL4 DNA binding domain or GAL4 activating domain.

To generate **pGDACP-190$^{1-765}$** and **pGBT9CP-190$^{1-765}$**, the corresponding fragment from CP190 cDNA was PCR-amplified with primers 5'-catgggtgaagtcaagtc-3' and 5'-ttcagatctttccaggttgtcaatgg-3' containing BglII site. This PCR product was cloned either in pGDA or pGBT9 cleaved with EcoRI (filled in with Klenow fragment) and BamHI.

To generate **pGDACP-190$^{1-608}$** and **pGBT9CP-190$^{1-608}$**, we used the same strategy, but the next pair of primers were 5'-catgggtgaagtcaagtc-3' and 5'-ttcagatctgccatcctccaaagcct-3' containing BglII site.

To generate **pGBT9CP190$^{1-125}$**, the EcoRI–NcoI (Klenow-filled) fragment from pGBT9CP-190$^{1-765}$ plasmid was cloned in pGBT9 cleaved with EcoRI–BamHI (filled in with Klenow fragment).

To generate **pGDACP190$^{1-125}$**, the EcoRI–NcoI fragment from pGBT9CP-190$^{1-765}$ plasmid was cloned in pGDA cleaved with EcoRI–NcoI.

To generate **pGBT9CP190$^{1-308}$** and **pGDACP190$^{1-308}$,** the corresponding fragment from CP190 cDNA was PCR-amplified with primers 5'-catgggtgaagtcaagtc-3' and 5'-gtggatccatgttctagttg-3' containing BamHI site. The PCR product was then cleaved with BamHI and cloned in corresponding vectors cleaved with EcoRI (filled in with Klenow fragment) and BamHI.

To generate **pGBTCP190$^{1-470}$** and **pGDACP190$^{1-470}$**, the corresponding fragment from CP190 cDNA was PCR-amplified with primers 5'-catgggtgaagtcaagtc-3' and 5'-

aaagatctgggcccagtagtattctct-3' containing BglII site. This PCR product was cleaved with BglII and cloned in corresponding vectors cleaved with EcoRI (filled in with Klenow fragment) and BamHI.

To generate **pGBT9CP190**$^{\mathbf{263\text{-}470}}$ and **pGDACP190**$^{\mathbf{263\text{-}470}}$, the corresponding fragment from CP190 cDNA was PCR-amplified with primers 5'-catgggtgaagtcaagtc-3' and 5'-aaagatctgggcccagtagtattctct-3' containing BglII site. The PCR product was then cleaved with BglII and EcoRI and cloned in corresponding vectors cleaved with EcoRI and BamHI.

pGBT9CP190$^{\mathbf{125\text{-}1096}}$ and **pGDACP190**$^{\mathbf{125\text{-}1096}}$ plasmids were generated by PCR-amplification from CP190 cDNA with primers 5'-cagatcttagtaaatatagctcctccttcg-3' containing BglII site and 5'-catggagaacgttaatcgccag-3'. The PCR product was cleaved with BglII and cloned in either pGBT9 or pGDA cleaved with EcoRI (filled in with Klenow fragment) and BamHI.

Constructs for GST Pull Down assay

pGex4T-1Su(Hw)$^{\mathbf{1\text{-}238}}$ was generated by cloning the BamHI–BamHI fragment from pET30a Su(Hw)$^{1\text{-}238}$ in pGex 4T-1 vector cleaved with BamHI.

pGex4T-1Su(Hw)$^{\mathbf{716\text{-}945}}$ and **pET32aSu(Hw)**$^{\mathbf{716\text{-}945}}$ were generated by PCR amplification of Su(Hw) cDNA with primers 5'-actcgagcagaccattacactagaaaac-3' and 5'-ctcgaggccgcaagctttctcttg-3' (both containing XhoI sites) and cloning of this fragment either in pGex4T-1 cleaved with XhoI or in pET32a cleaved with SalI and XhoI.

pET32aSu(Hw)$^{\mathbf{632\text{-}945}}$ and **pET32aSu(Hw)**$^{\mathbf{780\text{-}945}}$ were generated by PCR amplification of su(Hw) cDNA with primers 5'-actcgaggttaaacacatcagcgggctc-3' (for aa 632–945) or 5'-actcgagaccgatcctaaagaaatac-3' (for aa 780–945) on the 5' end and 5'-ctcgaggccgcaagctttctcttg-3' on the 3' end. The PCR products were cleaved with XhoI and cloned in pET32a cleaved with XhoI.

pGex2TMod-67.2$^{\mathbf{1\text{-}610}}$ was kindly provided by Maria Gause.

To generate **pET23aMod-67.2**$^{\mathbf{1\text{-}610}}$ Mod(mdg4)-67.2 cDNA from pCaSpeR2 (Golovnin et al., 2008) was cloned using *Eco*RI and filled in *Bam*HI in pET23a vector which was in turn digested by EagI, filled by Klenow, and than digested by EcoRI.

To generate **pET32aMod-67.2**$^{\mathbf{118\text{-}277}}$, the SacII–NcoI (Klenow-filled) fragment from pGDAMod-67.2 was cloned in pET32a cleaved with SmaI and NcoI.

pET32aMod-67.2$^{\mathbf{1\text{-}118}}$ was generated by PCR amplification with pair of primers 5'-accatgggaggcgcaggatcgttat-3' containing NcoI site and 5'-agatctaatggcggacgacgagcaattcag-3' containing BglII site. The PCR product was cleaved with NcoI and BglII and cloned in pET32a cleaved with the same enzymes.

Transgenic constructs

To prepare **pAc5.1Su(Hw)$^{1\text{-}238}$-FLAG**, the EcoRI–BamHI fragment from pGBTSu(Hw)$^{1\text{-}238}$ was cloned in the corresponding vector at EcoRI and BamHI sites.

To generate **pAc5-1 SuHw$^{1\text{-}945}$-FLAG** or **pAc5-1 SuHw$^{208\text{-}945}$-FLAG** plasmids, PCR fragment from SuHw cDNA with attached XhoI on 5' end (5' –CTCGAGATGAGTGCCTCCAAGGAGGGC- 3' for Su(Hw)$^{1\text{-}945}$and 5' – CTCGAGATGTCCTCAGGCAAGGGCAACTC - 3' for Su(Hw)$^{208\text{-}945}$) and BstXI on 3' end (5' –GTCCATGCTAGTGGATCTCGAAGCTTT- 3') was digested with these enzymes and cloned in the corresponding vector digested by XhoI and BstXI.

To express the transgenic constructs in flies, we generated vector **pUbqW** with ubiquitin-63E promoter and *miniwhite* gene as a marker. To prepare pUbqW, the fragment containing SV40 terminator from pUAST plasmid was recovered with XbaI and BamHI (both filled in with Klenow fragment) and cloned in pCaSpeR4 cleaved with PstI filled in with Klenow fragment (pCaSpeR4-SV40). The correct orientation of the fragment was verified by sequencing. Then a 1868-bp promoter region was PCR-amplified with primers 5'-agaattctgtcgccggaacgcag-3' and 5'-agaattcgcattttggattattctgcggg-3', both containing EcoRI site. The PCR product was recovered with EcoRI and cloned in pCaSpeR4-SV40 cleaved with the same enzyme. Its correct orientation was verified by sequencing.

To prepare the **P{w^{+};UbqW-Su(Hw)238-945-FLAG}** construct, the KpnI–NotI fragment from full-length cDNA of Su(Hw) protein fused to FLAG epitope was cloned in the pUbqW vector cleaved with KpnI and NotI. The resulting plasmid was cut with EagI and cloned with EagI–EagI fragment of the Su(Hw) C-terminal part fused with FLAG epitope.

To generate the **P{w^{+};UbqW-Su(Hw)1-945-FLAG}** construct, the KpnI–SacI fragment (filled in with Klenow fragment) containing full-length cDNA of Su(Hw) protein fused to FLAG epitope was cloned in pUbqW cleaved with KpnI and BamHI (filled in with Klenow fragment).

Preparation of **P{w^{+};UAS-Mod-67.2}** and **P{w^{+};UAS-ModΔQ}** was described previously (Golovnin et al., 2008).

To generate **P{w^{+};UAS-ModΔFLYWCH}**, Mod(mdg4)-67.2 cDNA was cleaved with BstEII (filled in with Klenow fragment) and Eco72I and self-ligated. The resulting ModΔFLYWCH cDNA was recovered with EcoRI and BamHI and cloned in the pUAST vector cleaved with EcoRI and BglII.

To generate **P{w^{+};UAS-ModΔCTD-FLAG}**, Mod-67.2 cDNA was cleaved with EcoRI and Eco72I and cloned in frame to FLAG epitope in the pAc5.1 vector digested with EcoRI and

BstXI (filled in with Klenow fragment). From the resulting plasmid, ModΔCTD fused with FLAG was recovered with EcoRI and BamHI and cloned in pUAST cleaved with EcoRI and BglII.

To generate **P{w^{+};UAS-46-33 ModΔQ}** fragment EcoRI-DraII from pSK Mod 46-33 (described in Golovin et al 2008) and fragment DraII-BamHI from pGDA ModΔQ were simultaneously ligated in pBluescript I1 SK+ digested with *Eco*RI and BamHI. Resulting Mod 46-33ΔQ cDNA was recover by EcoRI and BamHI enzymes and subclone to the pUAST vector deigested by EcoRI and BglII enzymes.

To generate **the Su(Hw)^{430} transgenic construct**, we used a 8-kb fragment containing the *yellow* gene and the cDNA *yellow* clone, which were kindly provided by P. Geyer. The 3-kb SalI–BamHI fragment containing the *yellow* regulatory region (*yr*) was subcloned into BamHI + XhoI-digested pGEM7 (yr plasmid). The yellow regulatory region included the body enhancer (between –1266 and –1963 bp relative to the *yellow* transcription start site) and wing enhancer (between –1863 and –2873 bp) (Geyer and Corces, 1987). The 5-kb BamHI–BglII fragment containing the coding region (yc) was subcloned into CaSpeR3 (C3-yc) vector. The 430-bp *gypsy* sequence containing the Su(Hw) binding region was PCR-amplified from the *gypsy* retrotransposon with primers 5'-ctggccacgtaataagtgtgcg-3' and 5'-gttgttggttggcacaccacaa-3'. After sequencing to confirm its identity, the product was inserted in pGEM7yr plasmid cleaved with Eco47III at –893 bp from the *yellow* transcription start site. The *yellow* regulatory region with 430-bp *gypsy* sequence was cloned into C3-yc cleaved with XbaI and BamHI.

References

1. Gdula, D.A. and Corces VG. (1997) Characterization of functional domains of the su(Hw) protein that mediate the silencing effect of mod(mdg4) mutations. *Genetics*, **145**, 153-161.

2. Mazur, A.M., Georgiev, P.G., Golovnin, A.K. (2005) The acid domain located at the C-terminus of the Su(Hw) protein represses transcription in the yeast two-hybrid system. *Dokl Biochem Biophys*., **400**,1-3.

3. Golovnin, A., Melnikova, L., Volkov, I., Kostuchenko, M., Galkin, A.V., Georgiev, P.(2008) 'Insulator bodies' are aggregates of proteins but not of insulators. *EMBO Rep.,* **9**, 440-445.

Supplementary Table

Table S1. Primer sequences used in PCR for ChIP analysis

Primer pair	Sequences
62D fw<br>62D rev	5' TTTGGGCTTGGTGAGAACAG 3'<br>5' TGATACCAGGCGAACAGAAATC 3'
50A fw<br>50A rev	5' ATACAAAGTGGTTTCAGCCAAGAAG 3'<br>5' TTGATAAATAGTCCAGCACGCATAC 3'
87E fw<br>87E rev	5' GGATGTTACA TTGAGAGTGCTTAGG 3'<br>5' TTTGCGTTTCGGCTGCTGTC 3'
1A2 fw<br>1A2 rev	5' ACCACACATCAGTCATCGTGT 3'<br>5' CTTCGTCTACCGTTGTGC 3'
Gypsy fw<br>Gypsy rev	5' TTCTCTAAAAAGTATGCAGCACTT 3'<br>5' CACGTAATAAGTGTGCGTTGA 3'
Ras fw<br>Ras rev	5' GAGGGATTCCTGCTCGTCTTCG 3'<br>5' GTCGCACTTGTTACCCACCATC 3'
15878 fw<br>15878 rev	5'GTGAACTCAATTTGCTGGCATC 3'<br>5'CGTTGTGTTCGCCGCTTTTG 3'
cg32333 fw<br>cg32333 rev	5' CAAAAAGTTGCCGGACGACCAAA 3'<br>5' CTACTTGCCAGCGCCAACCTT 3'
cg17090 fw<br>cg17090 rev	5’ CCTAAGGCAACCTGCAACATC 3’<br>5’ GCCGTGGCTGGAGTTATATG 3’
cg7943 fw<br>cg7943 rev	5’ GACTCCAAGTTCCACCAGCA 3’<br>5’ GCCTCTTCTCGCAGTACGA 3’
cg31472 fw<br>cg31472 rev	5’ CAATTCGAAGGATGAGCCAAGG 3’<br>5’ TCCAGCCAGTCTCGGAATAC 3’
Hml fw<br>Hml rev	5’ CAAGGGCATTAAACTGGAGCAC 3’<br>5’ CTCCTTGTACTGCACAGTTCC 3’
Adar fw<br>Adar rev	5' CTAGCAAGCCGATGATGAAGTTG 3'<br>5'GGTAAGGTTCACGCGAACCTAA 3'
cg43921 fw<br>cg43921 rev	5' CAGGACGATTGCGCAGCAAAG 3'<br>5' GCCCGCTATTGGTGGTGTTC 3'
